# The Protective Effect of Sevoflurane Conditionings Against Myocardial Ischemia/Reperfusion Injury: A Systematic Review and Meta-Analysis of Preclinical Trials in *in-vivo* Models

**DOI:** 10.3389/fcvm.2022.841654

**Published:** 2022-04-28

**Authors:** Bin Hu, Tian Tian, Pei-Pei Hao, Wei-Chao Liu, Ying-Gui Chen, Tian-Yu Jiang, Fu-Shan Xue

**Affiliations:** Department of Anesthesiology, Beijing Friendship Hospital, Capital Medical University, Beijing, China

**Keywords:** sevoflurane, preconditioning, postconditioning, myocardial ischemia/reperfusion injury, pre-clinical trials, infarct size, meta-analysis

## Abstract

**Objective:**

Myocardial ischemia/reperfusion injury (IRI) is a common and serious complication in clinical practice. Sevoflurane conditionings have been identified to provide a protection against myocardial IRI in animal experiments, but their true clinical benefits remain controversial. Here, we aimed to analyze the preclinical evidences obtained in animal models of myocardial IRI and explore the possible reasons for controversial clinical benefits.

**Methods:**

Our primary outcome was the difference in mean infarct size between the sevoflurane and control groups in animal models of myocardial IRI. After searching the databases of PubMed, Embase, Web of Science, and the Cochrane Library, a systematic review retrieved 37 eligible studies, from which 28 studies controlled comparisons of sevoflurane preconditioning (SPreC) and 40 studies controlled comparisons of sevoflurane postconditioning (SPostC) that were made in a pooled random-effects meta-analysis. In total, this analysis included data from 313 control animals and 536 animals subject to sevoflurane conditionings.

**Results:**

Pooled estimates for primary outcome demonstrated that sevoflurane could significantly reduce the infarct size after myocardial IRI whether preconditioning [weighted mean difference (WMD): −18.56, 95% CI: −23.27 to −13.85, *P* < 0.01; *I*^2^ = 94.1%, *P* < 0.01] or postconditioning (WMD: −18.35, 95% CI: −20.88 to −15.83, *P* < 0.01; *I*^2^ = 90.5%, *P* < 0.01) was performed. Interestingly, there was significant heterogeneity in effect size that could not be explained by any of the prespecified variables by meta-regression and stratified analysis. However, sensitivity analysis still identified the cardioprotective benefits of sevoflurane conditionings with robust results.

**Conclusion:**

Sevoflurane conditionings can significantly reduce infarct size in *in-vivo* models of myocardial IRI. Given the fact that there is a lack of consistency in the quality and design of included studies, more well-performed *in-vivo* studies with the detailed characterization of sevoflurane protocols, especially studies in larger animals regarding cardioprotection effects of sevoflurane, are still required.

## Introduction

Ischemic heart disease (IHD), especially acute myocardial infarction, is one of the leading causes of morbidity and mortality of the world populations (1). It is generally believed that timely and successful restoration of blood supply to the ischemic area is the most effective method to rescue the ischemic myocardium and improve clinical outcomes (2). In clinical practice, moreover, various treatment strategies for restoration of blood supply to the ischemic myocardium, such as primary percutaneous coronary intervention or coronary artery bypass grafting (CABG), have been widely used. As a “double-edged sword,” however, reperfusion of blood flow to ischemic myocardium can further worsen damage in the ischemic myocardium and even cause more deaths of cardiomyocytes, which are even attributable to about 50% of final myocardial infarct size (3, 4). This phenomenon is called as myocardial ischemia/reperfusion injury (IRI). For decades, abundant studies have been conducted, but the physiopathology mechanism underlying myocardial IRI remains elusive. The available literatures indicate that oxidative stress, apoptosis, and inflammation are extensively involved in the development of myocardial IRI (3, 5). Unfortunately, as an intractable clinical issue, there have been still no effective interventions against myocardial IRI (6). Hence, developing reasonable and practical countermeasures to provide protection against myocardial IRI are extremely essential.

Sevoflurane, an inhaled anesthetic with good pharmacological properties of stable induction and rapid recovery, is extensively applied for anesthesia of cardiac surgery in clinical practice (7). Early in 2007, a meta-analysis of small randomized clinical trials comparing inhaled anesthesia and total intravenous anesthesia (TIVA) has showed that both the sevoflurane and desflurane are associated with significant reductions of myocardial infarction (2.4 vs. 5.1%) and mortality (0.4 vs. 1.6%) (8). Furthermore, a RCT demonstrated a decreased 1-year mortality after CABG with cardiopulmonary bypass (CPB) in patients receiving sevoflurane anesthesia compared with TIVA (17.8 vs. 24.8%) (9). Similarly, animal experiments have also demonstrated that sevoflurane conditionings can provide a protection against myocardial IRI and the underlying mechanisms involve antioxidant, antiapoptosis, and anti-inflammation pathways (10, 11). However, a multicenter study found no difference in the occurrence of myocardial ischemia when comparing sevoflurane vs. TIVA in patients undergoing noncardiac surgery at risk for perioperative myocardial ischemia (12). These inconsistent findings have caused confusion of available evidence about cardioprotective benefits of sevoflurane conditionings.

Undeniably, there are still many differences between the preclinical and clinical studies due to the complexity of clinical situations. Actually, poor reporting of preclinical study protocols can potentially lead to unreliable experimental results and unnecessary clinical trials (13, 14). In contrast, well-designed animal studies can provide both the safe and effective information on relevant factors that may improve the possibility of success in future clinical trials (15). Furthermore, a systematic reviews of preclinical animal data may partly explain the underlying mechanisms of clinical diseases and indicate the directions of clinical treatments (16). Thus, well-designed experimental studies may provide a deep insight into the cardioprotective efficacy of sevoflurane and useful information on relevant factors that may influence outcomes. However, it must be noted that previous preclinical studies regarding cardioprotection of sevoflurane against myocardial IRI are usually small sample size and vary considerably in the quality of representation, resulting in a low power of evidence. Thus, this comprehensive systematic review and meta-analysis were conducted to pool the findings of previous animal studies regarding the cardioprotective effects of sevoflurane conditionings. The main aims of this analysis are to provide a comprehensive evaluation on cardioprotective efficacy of sevoflurane conditionings in *in-vivo* animal models of myocardial IRI and attempt to explore the possible reasons for controversial clinical benefits.

## Methods

### Search Strategy

We systematically searched the PubMed, Embase, Web of Science, and the Cochrane Library for evidence of the cardioprotective effects of sevoflurane conditionings in animal models of myocardial IRI published from the inception to July 2021. The search strategy was using selected keywords and the Medical Subject Headings (MeSH) terms where appropriately specific to each database (Supplementary Material 1). Furthermore, the reference of review articles, meeting abstracts, and comments for additional citations were searched for relevant studies.

### Inclusion and Exclusion Criteria

Studies that met the following criteria were included for further meta-analysis: (1) Sevoflurane vs. control treatment; (2) Infarct size was determined with blue dye (i.e., Evans blue)/2,3,5-Triphenyltetrazolium chloride (TTC) double staining and expressed as the percentage of infarct area over area at risk (AAR); (3) Animal models without risk factors of cardiovascular diseases (i.e., aging, diabetes, obesity, or hyperlipidemia); (4) Nonhuman setting; (5) Documented durations of ischemia and reperfusion; and (6) Documented times and dosage regimens of the sevoflurane conditionings. The *ex-vivo* or *in-vitro* studies assessing cardioprotective effects of sevoflurane conditionings were excluded.

### Data Extraction

Eligibility assessment was performed independently in an unblinded, standardized manner by Bin Hu and Tian Tian. Two reviewers extracted the data independently from included studies using predefined data fields and the discrepancies were resolved by consensus. The following information of each study is shown in Tables 1A,B (1) Studies' characteristics (i.e., first author's name, year of publication, countries, number of included animals, durations of ischemia, and reperfusion); (2) Animals' characteristics (i.e., species, sex, body weight/age, and anesthetics); (3) Intervention data (i.e., dosage and time of treatment), and (4) For each eligible study, if the associated information was present merely in figures, two reviewers (Bin Hu and Tian Tian) would use the Engauge Digitizer 11.1 to collect data from the statistical graphs independently (52). Then, the mean values would be adopted. If the complete data are still not available, the article with missing data will be excluded from data synthesis.

**Table 1A:** The characteristics of sevoflurane preconditioning (SPreC) studies included in the meta-analysis.

**Study ID**	**Countries**	**Species**	**Weight/Age**	**Anesthetics**	**Animal numbers**		**I/R duration (min)**	**Sevoflurane treatment**		**Infarct size/AAR**
					**Control**	**Sevoflurane**		**Doses**	**Times**	
Toller et al. (17)	America	Mongrel dog, M/F	26 ± 1 kg	Pentobarbital	9	8	60/180	1MAC	Administered for 30 min before I/R	Patent blue/TTC
Toller et al. (17)	America	Mongrel dog, M/F	26 ± 1 kg	Pentobarbital	9	7	60/180	1MAC	Administered for 30 min at 30 min before I/R	Patent blue/TTC
Obal et al. (18)	Germany	Wistar, Rats, M	397 ± 46 g	α-chloralose	9	10	25/120	2.0% (1MAC)	Administered for 2 cycles of 5 min at 10 min before I/R	Evans blue/TTC
Lange et al. (19)	Germany	Rabbit, NZW, M	NA	Pentobarbital	8	8	30/180	3.7% (1MAC)	Administered for 30 min at 60 min before I/R	Patent blue/TTC
Redel et al. (20)	Germany	C57BL/6, Mice, M	8–12 W	Pentobarbital	8	8	45/180	1MAC	Administered for 15 min at 30 min before I/R	Evans blue/TTC
Wang et al. (21)	China	SD, Rats, M	270–350 g	Pentobarbital	8	8	30/120	2.5% (0.9MAC)	Administered for 30 min at 15 min before I/R	Evans blue/TTC
Frassdorf et al. (22)	Netherlands	Wistar, Rats, M	380–420 g	S^+^-ketamine	6	6	25/120	2.4% (1MAC)	Administered for 5 min at 10 min before I/R	Evans blue/TTC
Frassdorf et al. (22)	Netherlands	Wistar, Rats, M	380–420 g	S^+^-ketamine	6	6	25/120	2.4% (1MAC)	Administered for 2 cycles of 5 min at 10 min before I/R	Evans blue/TTC
Frassdorf et al. (22)	Netherlands	Wistar, Rats, M	380–420 g	S^+^-ketamine	6	6	25/120	2.4 % (1MAC)	Administered for 3 cycles of 5 min at 10 min before I/R	Evans blue/TTC
Frassdorf et al. (22)	Netherlands	Wistar, Rats, M	380–420 g	S^+^-ketamine	6	6	25/120	2.4% (1MAC)	Administered for 6 cycles of 5 min at 10 min before I/R	Evans blue/TTC
Tosaka et al. (23)	Japan	SD, Rats, M	455 ± 33 g/ 14 ± 1 W	Sodium pentobarbital	7	7	30/120	2.0% (1MAC)	Administered for 15 min at 30 min before I/R	Patent blue/TTC
Xiao et al. (24)	China	SD, Rats, M	250–300 g	Pentobarbital	6	6	30/120	2.5%	Administered for 1 h at 24 h before I/R	Evans blue/TTC
Zhang et al. (25)	China	SD, Rats, M	130–180 g	Chloral hydrate	6	6	30/120	2.4%	Administered for 1 h at 24 h before I/R	Evans blue/TTC
Ma et al. (26)	China	SD, Rats, M	130–180 g	Pentobarbital	8	8	30/120	2.4%	Administered for 3 cycles of 5 min before I/R	Evans blue/TTC
Qiao et al. (27)	China	SD, Rats, M	270–320 g/ 9–10 W	Pentobarbital	6	6	30/120	2.5% (1MAC)	Administered for 2 h at 24 h before I/R	Evans blue/TTC
Zhao et al. (10)	China	WT, Mice, NA	6–7 W	2% Isoflurane	16	16	30/1440	2.0%	Administered for 3 cycles of 10 min at 15 min before I/R	Evans blue/TTC
Xie et al. (28)	China	SD, Rats, M	270–350 g	Pentobarbital	8	8	30/120	2.5% (1MAC)	Administered for 2 h at 24 h before I/R	Evans blue/TTC
Behmenburg et al. (29)	Germany	Wistar, Rats, M	303 ± 21g	Pentobarbital	8	8	25/120	1MAC	Administered for 1 h at 24 h before I/R	Evans blue/TTC
Behmenburg et al. (29)	Germany	Wistar, Rats, M	303 ± 21g	Pentobarbital	8	8	25/120	1MAC	Administered for 1 h at 48 h before I/R	Evans blue/TTC
Behmenburg et al. (29)	Germany	Wistar, Rats, M	303 ± 21g	Pentobarbital	8	8	25/120	1MAC	Administered for 1 h at 72 h before I/R	Evans blue/TTC
Behmenburg et al. (29)	Germany	Wistar, Rats, M	303 ± 21 g	Pentobarbital	8	8	25/120	1MAC	Administered for 1 h at 96 h before I/R	Evans blue/TTC
Behmenburg et al. (29)	Germany	Wistar, Rats, M	304 ± 36 g	Pentobarbital	8	8	25/120	1MAC	72 h after the first treatment (1 h), Administered second treatment for 1 h at 24 h before I/R	Evans blue/TTC
Behmenburg et al. (29)	Germany	Wistar, Rats, M	304 ± 36 g	Pentobarbital	8	8	25/120	1MAC	72 h after the first treatment (1 h), Administered second treatment for 1 h at 48 h before I/R	Evans blue/TTC
Behmenburg et al. (29)	Germany	Wistar, Rats, M	304 ± 36 g	Pentobarbital	8	8	25/120	1MAC	72 h after the first treatment (1 h), Administered second treatment for 1 h at 72 h before I/R	Evans blue/TTC
Behmenburg et al. (29)	Germany	Wistar, Rats, M	304 ± 36 g	Pentobarbital	8	8	25/120	1MAC	72 h after the first treatment (1 h), Administered second treatment for 1 h at 96 h before I/R	Evans blue/TTC
Liu et al. (30)	China	SD, Rats, M	200–220 g/ 6 W	Pentobarbital	6	6	30/360	2.4 %	Administered for 3 cycles of 5 min before I/R	Evans blue/TTC
Xie et al. (31)	China	C57BL/6 J, mice, M	8–10 W	2% Isoflurane	6	6	30/1,440	2.0%	Administered for 3 cycles of 10 min before I/R	Evans blue/TTC
Hong et al. (32)	China	SD, Rats, M	280 ± 50 g/ 10-12 W	Pentobarbital	10	10	30/120	2.4% (1MAC)	Administered for 2 h at 24 h before I/R	Evans blue/TTC

*The study ID is represented by last name of first author and year of publication. If study ID is repeated, it indicates that same study involves different intervention protocols. Please refer to the details of intervention protocols in table. M, male; W, weeks; MAC, minimum alveolar concentration; I/R, ischemia/reperfusion; TTC, 2,3,5-triphenyltetrazolium chloride*.

**Table 1B:** The characteristics of sevoflurane postconditioning (SPostC) studies included in the meta-analysis.

**Study ID**	**Countries**	**Species**	**Weight/Age**	**Anesthetics**	**Animal numbers**		**I/R duration (min)**	**Sevoflurane treatment**		**Infarct size/AAR**
					**Control**	**Sevoflurane**		**Doses**	**Times**	
Preckel et al. (33)	Germany	Rabbit, NZW	2.8–4.4 kg	Thiopental	10	10	30/120	3.7% (1MAC)	First 15 min of reperfusion	Evans blue/TTC
Obal et al. (34)	Germany	Wistar, Rats	486 ± 26.62 g	Pentobarbital	11	11	25/90	1.8% (0.7MAC)	First 15 min of reperfusion	Evans blue/TTC
Obal et al. (34)	Germany	Wistar, Rats	486 ± 26.62 g	Pentobarbital	11	11	25/90	2.4% (1MAC)	First 15 min of reperfusion	Evans blue/TTC
Obal et al. (34)	Germany	Wistar, Rats	486 ± 26.62 g	Pentobarbital	11	13	25/90	3.6% (1.5MAC)	First 15 min of reperfusion	Evans blue/TTC
Obal et al. (34)	Germany	Wistar, Rats	486 ± 26.62 g	Pentobarbital	11	12	25/90	4.8% (2MAC)	First 15 min of reperfusion	Evans blue/TTC
Obal et al. (35)	Germany	Wistar, Rats	490 ± 38.42 g	Chloralose	7	8	25/90	2.4% (1MAC)	First 2 min of reperfusion	Evans blue/TTC
Obal et al. (35)	Germany	Wistar, Rats	490 ± 38.42 g	Chloralose	7	8	25/90	2.4% (1MAC)	First 5 min of reperfusion	Evans blue/TTC
Obal et al. (35)	Germany	Wistar, Rats	490 ± 38.42 g	Chloralose	7	7	25/90	2.4% (1MAC)	First 10 min of reperfusion	Evans blue/TTC
Obal et al. (18)	Germany	Wistar, Rats, M	397 ± 46 g	α-chloralose	9	10	25/120	2.0% (1MAC)	First 2 min of reperfusion	Evans blue/TTC
Huhn et al. (36)	Netherlands	Wistar, Rats, M	250–350 g	S-ketamine	9	11	25/120	2.4% (1MAC)	Starting at 1 min before reperfusion and continuing into first 5 min of reperfusion	Evans blue/TTC
Redel et al. (20)	Germany	C57BL/6, Mice, M	8–12 W	Pentobarbital	8	8	45/180	1MAC	Starting at 3 min before reperfusion and continuing into first 15 min of reperfusion	Evans blue/TTC
Tosaka et al. (23)	Japan	SD, Rats, M	455 ± 33 g/14 ± 1W	Pentobarbital	7	7	30/120	2.0% (1MAC)	Starting at 3 min before reperfusion and continuing into first 5 min of reperfusion	Patent blue/TTC
Drenger et al. (37)	America	SD, Rats, M	310–340 g/3 months	Ketamine and xylazine	10	8	30/180	2.4% (1MAC)	First 5 min of reperfusion	Evans blue/TTC
Tai et al. (38)	China	SD, Rats, M	250–300 g	Pentobarbital	7	7	30/120	1MAC	First 5 min of reperfusion	Evans blue/TTC
Chen et al. (39)	China	Japanese White Rabbits, M/F	2.5–3.0 kg	Ketamine and xylazine	8	8	15/120	1.0%	First 5 min of reperfusion	Evans blue/TTC
Chen et al. (39)	China	Japanese White Rabbits, M/F	2.5–3.0 kg	Ketamine and xylazine	8	8	15/120	2.0%	First 5 min of reperfusion	Evans blue/TTC
Chen et al. (39)	China	Japanese White Rabbits, M/F	2.5–3.0 kg	Ketamine and xylazine	8	8	15/120	4.0%	First 5 min of reperfusion	Evans blue/TTC
Chen et al. (39)	China	Japanese White Rabbits, M/F	2.5–3.0 kg	Ketamine and xylazine	7	8	30/120	1.0%	First 5 min of reperfusion	Evans blue/TTC
Chen et al. (39)	China	Japanese White Rabbits, M/F	2.5–3.0 kg	Ketamine and xylazine	7	8	30/120	2.0%	First 5 min of reperfusion	Evans blue/TTC
Chen et al. (39)	China	Japanese White Rabbits, M/F	2.5–3.0 kg	Ketamine and xylazine	7	8	30/120	4.0%	First 5 min of reperfusion	Evans blue/TTC
Chen et al. (39)	China	Japanese White Rabbits, M/F	2.5–3.0 kg	Ketamine and xylazine	5	7	60/120	1.0%	First 5 min of reperfusion	Evans blue/TTC
Chen et al. (39)	China	Japanese White Rabbits, M/F	2.5–3.0 kg	Ketamine and xylazine	5	6	60/120	2.0%	First 5 min of reperfusion	Evans blue/TTC
Chen et al. (39)	China	Japanese White Rabbits, M/F	2.5–3.0 kg	Ketamine and xylazine	5	5	60/120	4.0%	First 5 min of reperfusion	Evans blue/TTC
Xu et al. (40)	China	SD, Rats, M	130–180 g/6 W	Pentobarbital	8	8	30/120	2.4%	First 5 min of reperfusion	Evans blue/TTC
Li et al. (41)	China	SD, Rats, M	330 ± 5 g/3–4 months	Pentobarbital	6	6	30/120	3.0%	First 5 min of reperfusion	Evans blue/TTC
Zhang et al. (42)	China	SD, Rats, M	250–300 g/8 W	Chloral hydrate	6	6	30/120	2.4%	First 5 min of reperfusion	Evans blue/TTC
Stumpner et al. (43)	Germany	C57BL/6, Mice, M	8–12 W	Pentobarbital	7	7	45/180	1MAC	Starting at 3 min before reperfusion and continuing into first 15 min of reperfusion	Evans blue/TTC
Gao et al. (44)	China	C57BL/6, Mice, M	7–8 W	Pentobarbital	7	7	45/120	2.0%	First 15 min of reperfusion	Evans blue/TTC
Lin et al. (45)	China	SD, Rats, M	300–350 g	Pentobarbital	6	6	30/90	2.0%	Administered for 15 min before reperfusion	Evans blue/TTC
Li et al. (46)	China	SD, Rats, M	330 ± 8 g/ 3–4 months	Pentobarbital	6	6	30/120	1MAC	First 5 min of reperfusion	Evans blue/TTC
Li et al. (46)	China	SD, Rats, M	330 ± 8 g/ 3–4 months	Pentobarbital	6	6	30/120	2MAC	First 5 min of reperfusion	Evans blue/TTC
Qiao et al. (47)	China	SD, Rats, M	300 ± 50 g/9–12 W	Pentobarbital	8	8	30/120	1MAC	First 15 min of reperfusion	Evans blue/TTC
Qi et al. (11)	China	C57BL/6, Mice, M	NA	Ketamine plus xylazine	10	10	30/360	3.4%	First 5 min of reperfusion	Azo-blue/ TTC
Qi et al. (11)	China	C57BL/6, Mice, M	NA	Ketamine plus xylazine	10	10	30/7 days	3.4%	First 5 min of reperfusion	Azo-blue/ TTC
Huang et al. (48)	China	C57BL/6, Mice, M	20–30 g/ 10–12 W	Pentobarbital	10	15	30/120	2.4%	First 5 min of reperfusion	Evans blue/TTC
Tan et al. (49)	China	SD, Rats, M	250 ± 50 g/ 7–8 W	Pentobarbital	6	6	30/120	1.0%	First 5 min of reperfusion	Azo-blue/ TTC
Tan et al. (49)	China	SD, Rats, M	250 ± 50 g/ 7–8 W	Pentobarbital	6	6	30/120	2.0%	First 5 min of reperfusion	Azo-blue/TTC
Tan et al. (49)	China	SD, Rats, M	250 ± 50 g/ 7–8 W	Pentobarbital	6	6	30/120	4.0%	First 5 min of reperfusion	Azo-blue/ TTC
Yu et al. (50)	China	SD, Rats, M	220–250 g	Urethane	3	3	30/120	2.5%	Starting 1 min before reperfusion and continuing into first 10 min of reperfusion	Evans blue/TTC
Gao et al. (51)	China	C57BL/6, Mice, M	7–8 W	Pentobarbital	7	7	45/120	2.0%	First 15 min of reperfusion	Evans blue/TTC

***Note:**The study ID is represented by last name of first author and year of publication. If study ID is repeated, it indicates that same study involves different intervention protocols. Please refer to the details of intervention protocols in table. M, male; W, weeks; MAC, minimum alveolar concentration; TTC, 2,3,5-triphenyltetrazolium chloride*.

### Quality Assessment

The quality of included studies was assessed and graded by two reviewers (Bin Hu and Tian Tian) based on the published criteria for animal experiments using the “Animal Research: Reporting of *in vivo* Experiments” (ARRIVE) guidelines 2.0 and a 12-item quality score (15, 53). The quality of study was assessed independently from data extraction and between assessors in an unblinded, standardized manner by two reviewers (Bin Hu and Tian Tian). Discrepancies were resolved by consensus or by another reviewer (Pei-Pei Hao), if necessary.

### Statistical Analysis

For statistical comparisons, sevoflurane interventions were divided into the two main groups: preconditioning (SPreC), in where sevoflurane was given at any time before the onset of ischemia and postconditioning (SPostC), in where sevoflurane was administrated during ischemia or at the beginning of reperfusion. The weighted (unstandardized) mean difference (WMD) in the infarct size between the sevoflurane and control groups was used as primary outcome to determine the cardioprotective efficacy of sevoflurane conditionings. The number of animals in the control group was corrected based on the number of comparisons for each series of experiments (*n*/number of comparisons) (54). To account for anticipated heterogeneity, effect sizes were pooled by using a random-effects meta-analysis, which considers the within-study and between-study variability and weights each study accordingly (15). The extent of heterogeneity among studies was assessed with the Cochran's *Q* test and further quantified by *I*^2^ statistics. Potential publication bias was assessed by visual inspection of a funnel plot for asymmetry and further detected by the Egger's and Begg's tests. If significant heterogeneity (*P*< *0.1*) was found across the studies, sensitivity analysis was conducted by removing each study in turn. Furthermore, the univariate meta-regression and stratified analysis (i.e., countries, species, ischemia duration, reperfusion duration, and timing regimens of interventions) were proposed to explore the potential sources of heterogeneity for primary outcome. The STATA version 16.0 statistical software (STATA Corporation, College Station, Texas, USA) was applied for analysis of all the data and the GraphPad Prism for Windows (version 9, GraphPad Software Incorporation, San Diego, California, USA) was used for production of all the figures. A *P*-value <0.05 was considered as statistically significant.

## Results

### Study Selection

**Figure 1:**
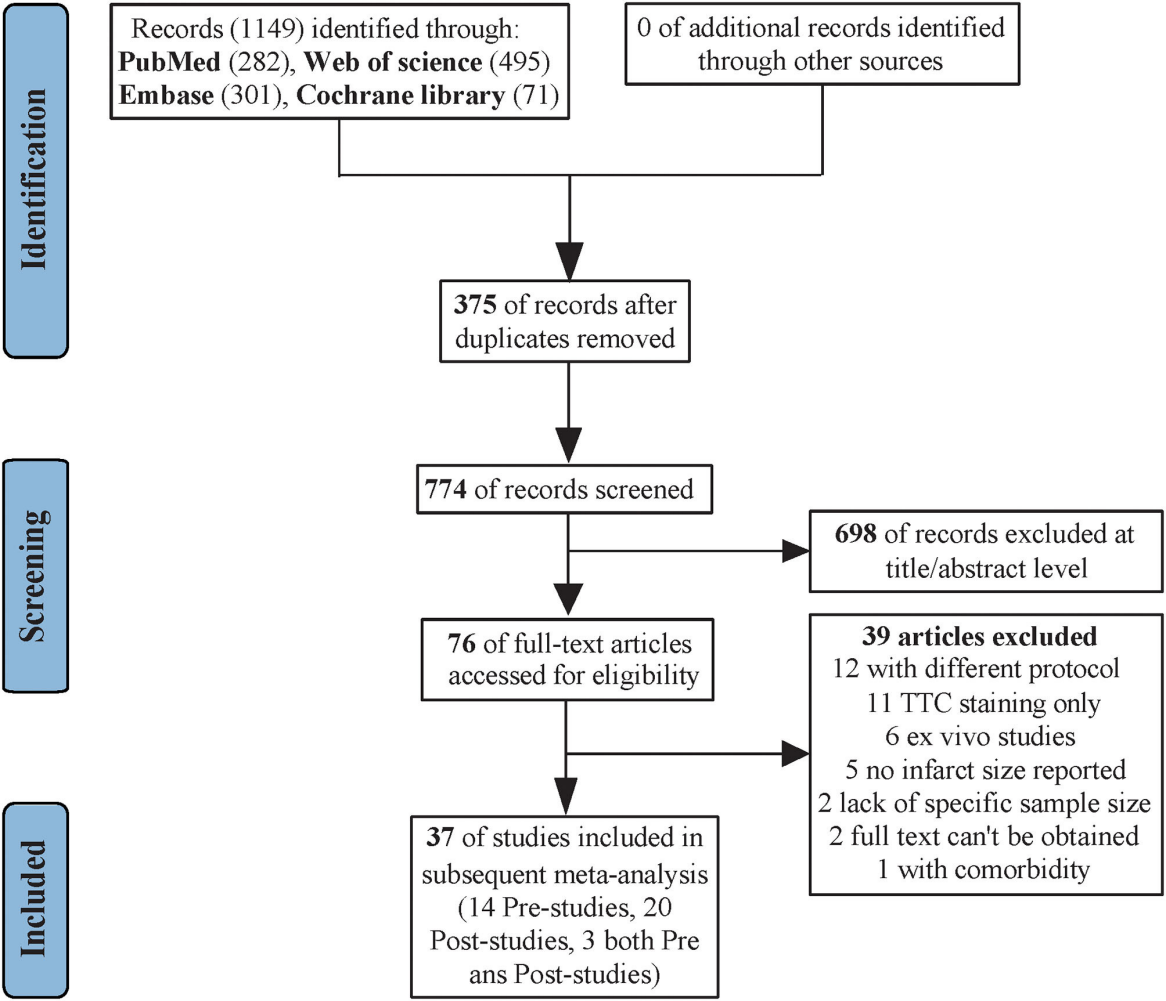
Flow diagram of the study selection process.

Our initial search identified 1,149 records, including 375 duplicate reports. In total, 774 reports underwent title and abstract screening, which resulted in 698 exclusions. The remaining 76 reports were retrieved for evaluation of detailed full text. Eventually, 39 articles were further excluded; of them, 12 articles were due to difference of study protocols, 11 articles had TTC staining only, 6 articles were *ex-vivo* studies, 5 articles did not report infarct size, 2 articles were no sample size per group, 2 articles could not acquire full text, and 1 article used animal model with the risk factors of cardiovascular diseases (Figure 1). As a result, 37 literatures [(14 SPreC studies (10, 17, 19, 21, 22, 24–32), 20 SPostC studies (11, 33–51), and 3 both the SPreC and SPostC studies (18, 20, 23)] met our selection criteria and were included in the analysis. Of 37 included studies, 7 studies provided the associated information of infarct size merely in figures, including 3 SPreC studies (10, 30, 31) and 4 SPostC studies (37, 45, 49, 50). Thus, the Engauge Digitizer 11.1 was used to collect data from the statistical graphs and obtained the mean values.

### Meta-Analysis

From the 37 included studies, we extracted data on 68 controlled comparisons of sevoflurane conditionings in the models of myocardial IRI. These were split into 28 comparisons assessing SPreC and 40 comparisons evaluating SPostC. In total, our analysis included data from 313 control animals and 536 animals receiving sevoflurane conditionings with statistical correction.

**Figure 2:**
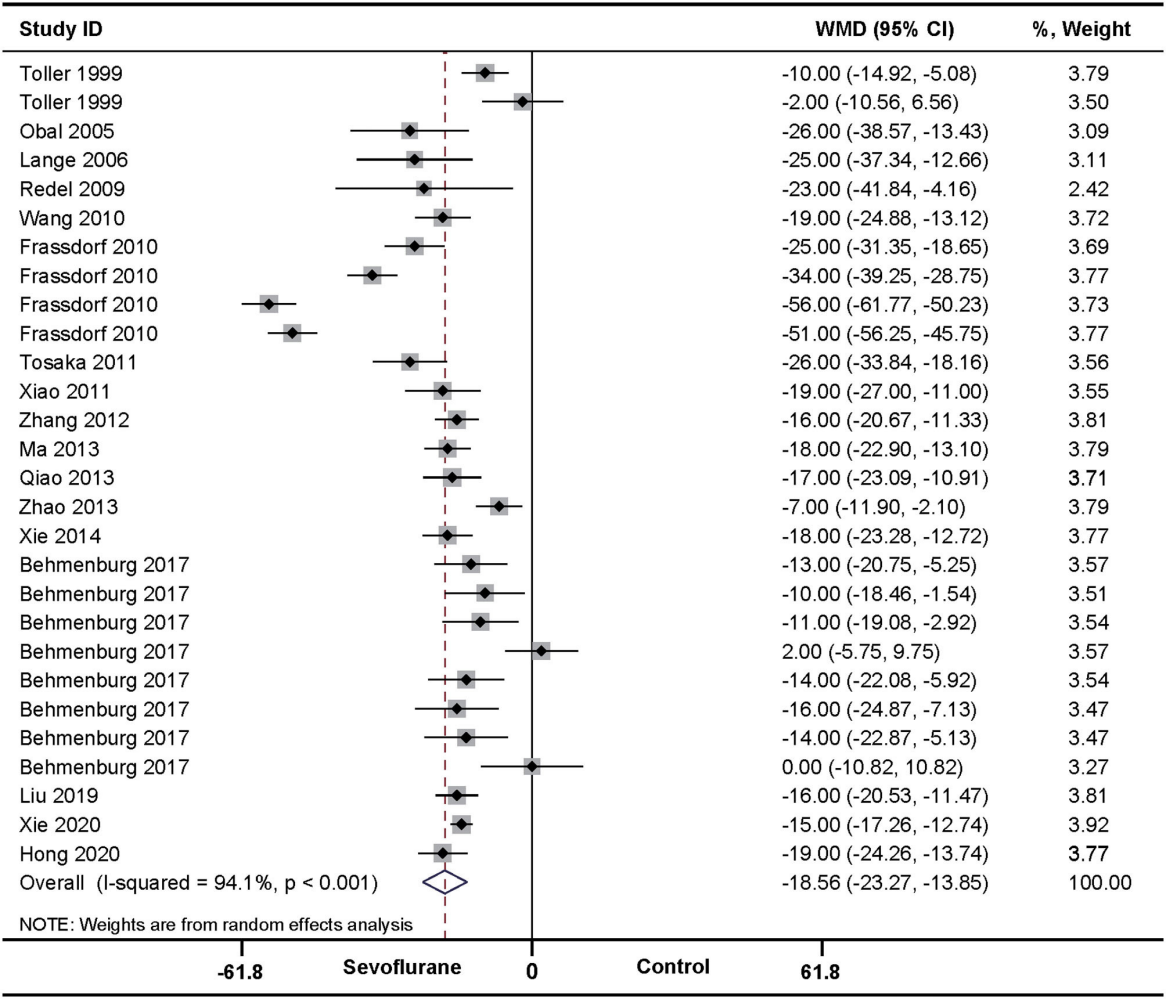
Pooled estimates of infarct size for sevoflurane preconditioning (SPreC) vs. control. Forest plots of meta-analysis of SPreC on myocardial infarct size pooled using a random-effects meta-analysis. The study ID is represented by last name of first author and year of publication. If a study ID is repeated, it indicates that same study involves different intervention protocols. Please refer to the details of intervention protocols in Table 1A.

In the pooled analysis using a random-effects model, SPreC compared with control treatment significantly diminished the infarct size (WMD: −18.56, 95% CI: −23.27 to −13.85, *P* < 0.01). Significant heterogeneity among studies was observed (*I*^2^ = 94.1%, *P* < 0.01) (Figure 2). By systematically excluding each study, the infarct size was still significantly reduced with SPreC over control treatment (Supplementary Table 1). Meta-regression (Table 2 and Supplementary Material 2) and stratified analysis (Table 3 and Supplementary Material 3) did not unmask significant impacts of prespecified covariates (i.e., countries, species, ischemia duration, reperfusion duration, and timing regimens of treatments) on the infarct size sparing benefit of SPreC. Meta-regression showed that country difference might be a source of significant heterogeneity (*P*< *0.0*5). Stratified analysis by countries showed that there was still significant heterogeneity between the groups. Especially, compared to other countries, Netherlands showed a large WMD and a wide 95% CI.

**Table 2:** The univariate meta-regression analyses to determine potential sources of heterogeneity for infarct size sparing in the included studies of SPreC and SPostC.

**Covariates**	**Infarct size (SPreC)**				**Infarct size (SPostC)**			
	**Coefficients**	**95%CI**	***P*** **-value**		**Coefficients**	**95%CI**	***P*** **-values**	
**Countries**	−7.17	−13.68, −0.66	0.03		−0.77	−6.63, 5.09	0.79	
**Species**	1.78	−5.09, 8.65	0.60		2.38	−2.39, 7.15	0.32	
**Ischemia duration**	−5.48	−15.92, 4.97	0.29	*I2* = 85.46%	−0.72	−8.06, 6.62	0.84	*I^2^* = 85.37%
**Reperfusion duration**	−3.96	−22.44, 14.52	0.66	*P* = 0.97	8.21	−3.53, 19.94	0.16	*P* = 0.13
**Timing regimen of treatment**	9.50	−2.70, 21.71	0.12		−3.86	−12.34, 4.62	0.36	
**Cycles of administration**	−1.60	−10.64, 7.44	0.72		–	**–**	–	
**Administration before reperfusion**	**–**	**–**	**–**		−1.13	−9.26, 6.99	0.78	

*Meta-regression provided valuable information regarding the interactions between the continuous covariates and treatment effect of SPreC or SPostC in limiting the infarct size and may explore the source of heterogeneity. P < 0.05 indicates the potential source of heterogeneity*.

**Table 3:** Stratified analysis of pooled estimates for infarct size sparing in the included studies of SPreC and SPostC.

**Pooled estimates**	**No. of studies**	**WMD (95%CI)**	***P*** **values**	**Heterogeneity**
**SPreC**				
**Countries**				
America Germany	2 11	−6.80 (−14.48, 0.88) −12.62 (−17.54, −7.71)	*P* = 0.08 *P* <0.01	*I^2^* = 60%, *P* = 0.11 *I^2^* = 66%, *P* <0.01
China	10	−16.07 (−18.20, −13.94)	*P* <0.01	*I^2^* = 49%, *P* = 0.04
Netherlands	4	−41.54 (−55.16, −27.92)	*P* <0.01	*I^2^* = 96%, *P* <0.01
Japan	1	−26.00 (−33.84, −18.16)	*P* <0.01	–
**Species**				
Dog Rat	2 22	−6.80 (−14.48, 0.88) −19.99 (−25.84, −14.15)	*P* = 0.08 *P* <0.01	*I^2^* = 60%, *P* = 0.11 *I^2^* = 94%, *P* <0.01
Rabbit	1	−25.00 (−37.34, −12.66)	*P* <0.01	–
Mice	3	−12.63 (−19.76, −5.50)	*P* <0.01	*I^2^* = 79%, *P* <0.01
**Ischemia duration**				
<30min	13	−20.80 (−31.53, −10.07)	*P* <0.01	*I^2^* = 96%, *P* <0.01
≥30min	15	−15.91 (−18.41, −13.41)	*P* <0.01	*I^2^* = 67%, *P* <0.01
**Reperfusion duration**				
≤ 2h	21	−20.18 (−26.39, −13.97)	*P* <0.01	*I^2^* = 95%, *P* <0.01
>2h	7	−12.42 (−16.63, −8.22)	*P* <0.01	*I^2^* = 74%, *P* <0.01
**Timing regimen of treatment**				
≤ 30min	15	−23.53 (−31.11, −15.96)	*P* <0.01	*I^2^* = 97%, *P* <0.01
>30min	13	−13.31 (−16.61, −10.02)	*P* <0.01	*I^2^* = 64%, *P* <0.01
**Cycles of administration**				
Yes No	12 16	−22.42 (−31.54, −13.29) −15.40 (−18.91, −11.90)	*P* <0.01 *P* <0.01	*I^2^* = 97%, *P* <0.01 *I^2^* = 75%, *P* <0.01
**SPostC**				
**Countries**				
America Germany	1 11	−9.00 (−9.93, −8.07) −22.50 (−28.19, −16.80)	*P* <0.01 *P* <0.01	– *I^2^* = 70%, *P* <0.01
China Netherlands Japan	26 1 1	−17.12 (−20.12, −14.11) −18.00 (−26.35, −9.65) −25.00 (−32.37, −17.63)	*P* <0.01 *P* <0.01 *P* <0.01	*I^2^* = 89%, *P* <0.01 – –
**Species**				
Rat	23	−19.98 (−23.27, −16.70)	*P* <0.01	*I^2^* = 91%, *P* <0.01
Rabbit	10	−12.43 (−17.68, −7.18)	*P* <0.01	*I^2^* = 53%, *P* = 0.02
Mice	7	−19.59 (−26.34, −12.84)	*P* <0.01	*I^2^* = 95%, *P* <0.01
**Ischemia duration**				
<30min	12	−18.04 (−24.23, −11.86)	*P* <0.01	*I^2^* = 80%, *P* <0.01
≥30min	28	−18.40 (−21.25, −15.55)	*P* <0.01	*I^2^* = 92%, *P* <0.01
**Reperfusion duration**				
≤ 2h	35	−17.70 (−20.18, −15.22)	*P* <0.01	*I^2^* = 82%, *P* <0.01
>2h	5	−23.32 (−34.83, −11.81)	*P* <0.01	*I^2^* = 98%, *P* <0.01
**Timing regimen of treatment**				
≤ 5min	27	−19.61 (−23.24, −15.97)	*P* <0.01	*I^2^* = 93%, *P* <0.01
>5min	13	−15.04 (−18.33, −11.76)	*P* <0.01	*I^2^* = 73%, *P* <0.01
**Administration before reperfusion**				
Yes No	13 27	−18.52 (−23.45, −13.59) −18.28 (−21.55, −15.01)	*P* <0.01 *P* <0.01	*I^2^* = 83%, *P* <0.01 *I^2^* = 92%, *P* <0.01

*Stratified analysis investigated whether particular categorical covariates explain any of the heterogeneity of treatments between studies*.

**Figure 3:**
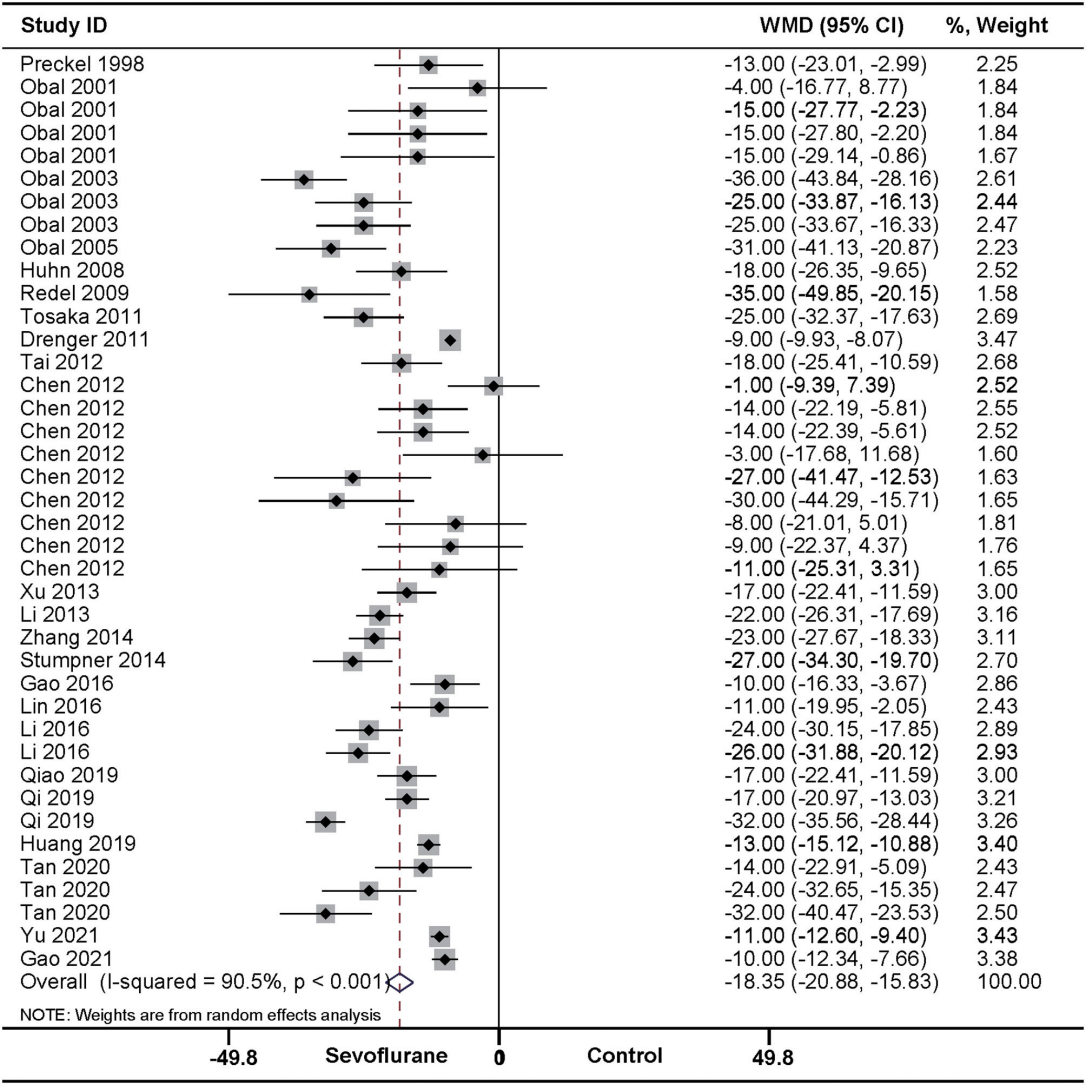
Pooled estimates of infarct size for sevoflurane postconditioning (SPostC) vs. control. Forest plots of meta-analysis of SPostC on myocardial infarct size pooled using a random-effects meta-analysis. The study ID is represented by last name of first author and year of publication. If a study ID is repeated, it indicates that same study involves different intervention protocols. Please refer to the details of intervention protocols in Table 1B.

In accordance with the above effect of SPreC, compared with control treatment, SPostC also significantly diminished the infarct size (WMD: −18.35, 95% CI: −20.88 to −15.83, *P* < 0.01). Again, significant heterogeneity among studies was observed (*I*^2^ = 90.5%, *P* < 0.01) (Figure 3). Sensitivity analysis by systematically removing each study provided a consistent estimation for the infarct size sparing benefit of SPostC (Supplementary Table 1). Similarly, meta-regression (Table 2 and Supplementary Material 2) and stratified analysis (Table 3 and Supplementary Material 3) showed no relationship between the prespecified covariates and pooled estimates.

### Risk of Bias

**Figure 4:**
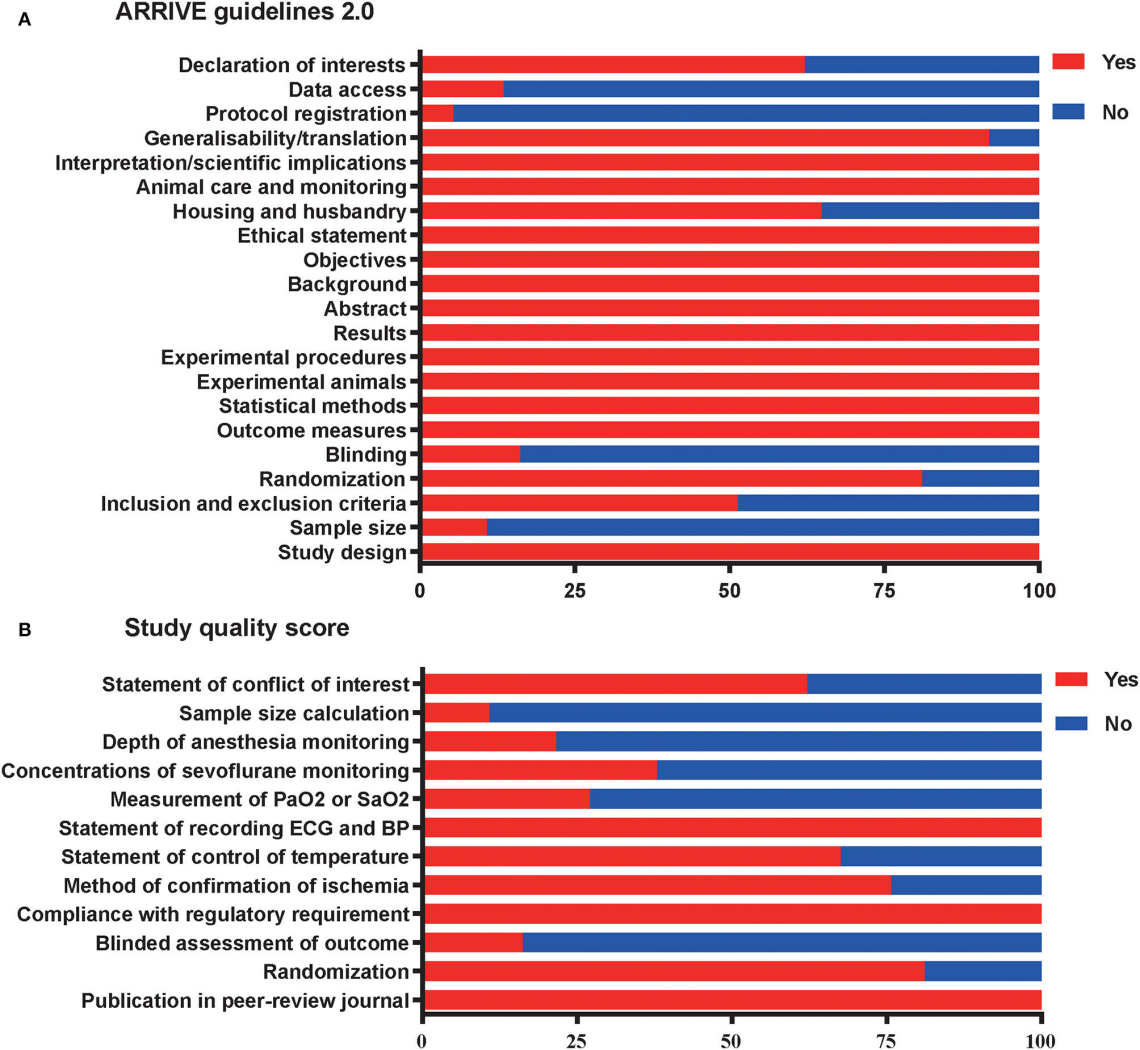
Reporting of study quality indicators. Study quality was assessed using the Animal Research: Reporting of *in vivo* Experiments (ARRIVE) guidelines 2.0 on reporting **(A)** and a 12-item quality score **(B)**. Values are expressed as the percentage of studies reporting each quality indicator.

**Figure 5:**
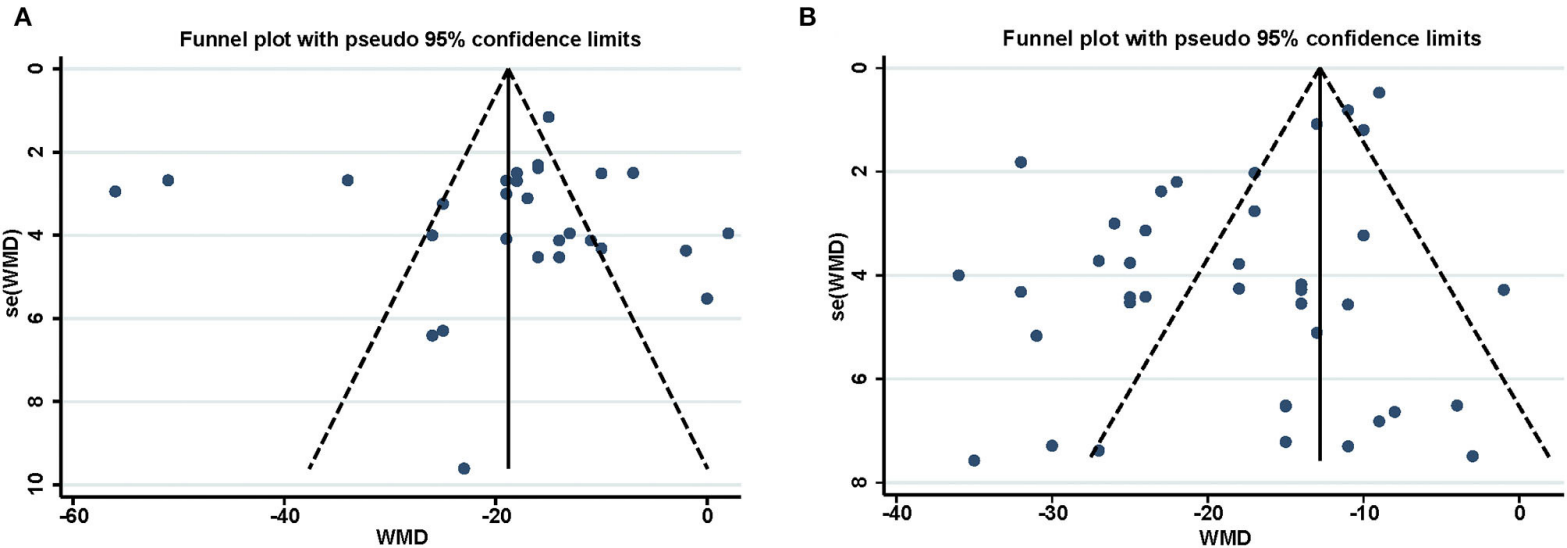
Funnel plot for assessment of publication bias for the infarct size in SPreC **(A)** and SPostC **(B)**. The vertical line represents the mean effect size. The plots were assessed visually, with further analysis of publication bias performed using the Egger's and Begg's tests.

Reports achieved the median ARRIVE guidelines 2.0 score of 16 out of 21 (interquartile range, 15–17) (Figure 4A) and a median 12-item quality score of 7 out of 12 (interquartile range, 6–8) (Figure 4B). The publication bias for the included studies was detected both visually (funnel plot) and mathematically (Egger's and Begg's tests) by plotting the effect size (WMD) of each controlled comparison against its SD for the SPreC and SPostC groups. Absence of publication bias was identified by the Egger's (*P* = 0.92) and Begg's (*P* = 0.78) tests in spite of an asymmetrical funnel plot for the SPreC group (Figure 5A). However, for the SPostC group, visual inspection of funnel plots showed that many studies fell outside of 95% CI (Figure 5B). Particularly, publication bias was identified by Egger's test (*P* < 0.01), despite not indicated by Begg's test (*P* = 0.28).

The meta-analysis demonstrated that the included studies were particularly poor in some important areas of experimental design. For example, with respect to the ARRIVE guidelines 2.0, only 11% of included studies reported a sample size calculation, 16% of included studies performed a blinded assessment of outcomes, 5% of included studies declared the protocol registration, and 14% of included studies reported data access (Figure 4A). Regarding the 12-item quality score, moreover, only 27% of studies reported measurement of partial pressure of oxygen (PaO_2_) or oxygen saturation (SaO_2_), 38% of studies monitored the sevoflurane concentrations, and 22% of studies monitored the anesthesia depth (Figure 4B).

## Discussion

The main findings of this comprehensive systematic review and meta-analysis are that compared with control treatment, both the SPreC and SPostC can provide a significant protection against myocardial IRI, shown by a significant infarct size sparing. Furthermore, the robust effect is comparable whether sevoflurane conditioning is carried out before the onset of ischemia, during ischemia, or at the time of reperfusion based on *in-vivo* data over 800 animals. In addition, the benefits of sevoflurane conditionings on myocardial IRI are not significantly affected by countries, species, duration of ischemia or reperfusion, and timing regimens of drug administration.

Myocardial IRI is a common and serious complication in clinical practice and has aroused extensive attention. Whether with the help of mechanical or pharmacological interventions, it is essential to reestablish coronary blood flow to limit myocardial damage following ischemia (55). The pathogenesis underlying myocardial IRI is extraordinarily complex and multifactorial. Among the etiological factors of myocardial IRI, oxidative stress is a vital mechanism, which initiates at the onset of reperfusion and triggers subsequent series of pathophysiological processes. Essentially, oxidative stress is characterized as severe imbalance between exaggerated reactive oxygen species (ROS) generation and inhibited antioxidant defense systems. Thus, ROS is considered as major determinants for adverse ventricular remodeling *via* promoting myocardial interstitial fibrosis, cardiomyocyte hypertrophy, and induction of cell death (56). Meanwhile, excessive oxidative stress causes mitochondrial permeability transition pore (mPTP) opening and cytochrome C releasing, subsequently triggering the intrinsic apoptosis by activation of caspase-9/3 signaling pathway (57). After reperfusion injury, oxidative stress and apoptosis collectively contribute to the substantial loss of cardiomyocyte and consequently enlarged infarct area. These pathological mechanisms provide the rationale for therapeutic strategies targeted against myocardial IRI.

As sevoflurane is an inhalational anesthetic most commonly used in clinical practice, its cardioprotection has attracted wide concern in the past decades. A recent meta-analysis elaborates the importance of various mechanisms involved in preconditioning and postconditioning of halogenated gases for cardioprotection (58). Several signaling molecules and pathways have been shown to be involved in sevoflurane-induced cardioprotection, but detailed mechanisms are not fully understood yet. To better understand the cardioprotection of sevoflurane conditionings, this analysis also included the *in-vivo* evidence regarding the potential mechanisms of sevoflurane conditioning reported in available literatures (Supplementary Table 2). Substantial literatures indicate that cardioprotection of sevoflurane conditionings is closely related to its antioxidant, antiapoptosis, and anti-inflammation properties. For example, Zhao et al. (10) established mice model of myocardial IRI *in vivo* and adult mouse cardiomyocytes model of simulated ischemia and reoxygenation (SI/R) *in vitro* and demonstrated that SPreC compared with control treatment decreased superoxide generation by 43.6%. Furthermore, this antioxidant property was largely retained in the AMP-activated protein kinase dominant-negative (AMPK-DN) mice, but completely abolished in caveolin-3 knockout (Cav-3KO) mice. These results suggest that SPreC-mediated attenuation of superoxide generation is highly dependent on Cav-3 and only partially dependent on the AMPK signaling axis. In addition, this study showed that SPreC compared with control treatment significantly decreased caspase-3 activity, indicated inhibition of cardiomyocyte apoptosis involved in the cardioprotection of SPreC. Previous study had identified that microRNAs (miRNAs) were important targets for regulating myocardial reperfusion damage and were the valuable biomarkers of myocardial injury (5). The study of Tan et al. (49) on a rat model of myocardial IRI *in vivo* demonstrated that SPostC compared with control treatment significantly decreased myocardial malondialdehyde level, but increased myocardial glutathione and superoxide dismutase activities, indicating that SPostC can inhibit oxidative stress in ischemia/reperfused myocardium by activating miR-203. Besides, this study also showed that SPostC decreased serum proinflammation factors and cardiomyocyte apoptosis. Similarly, Qi et al. (11) found that SPostC significantly inhibited oxidative stress and apoptosis to provide a protect against myocardial IRI by upregulating miR-145 expression and downregulating granzyme K expression.

It must be emphasized that several interesting phenomena about cardioprotection of SPreC and SPostC deserve special attention. Qiao et al. (47) demonstrated that cardioprotection of SPostC is age dependent, i.e., a significant cardioprotection is achieved in young animals but not in old ones. Available evidence indicates that this age-dependent cardioprotection of SPostC is at least associated with the inability to activate Akt and Erk1/2 in old animals (41). Furthermore, Obal et al. (18) found that a combination of SPreC with SPostC can provide an additive protection against myocardial IRI, which is partly mediated by opening mitochondrial adenosine triphosphate-dependent potassium (mK_ATP_) channels. In addition, various risk factors of cardiovascular diseases, such as obesity, hyperlipidemia, and diabetes, can significantly attenuate or even abolish the cardioprotective effects of sevoflurane conditionings (26, 50, 59). As a main aim of this analysis was to determine the cardioprotective effects of sevoflurane conditionings, the preclinical studies performed on animals with the risk factors of cardiovascular diseases were specifically excluded from this analysis. Nevertheless, it is advisable to consider the risk factors of cardiovascular diseases when determining the optimum protocols of sevoflurane conditionings.

Although many *in-vivo* experiments have shown that sevoflurane conditionings can provide an infarct size sparing, clinical trials in humans have yielded variable results regarding cardioprotection of sevoflurane conditionings. Several studies demonstrate that sevoflurane has the potential benefits of cardioprotection in patients undergoing coronary artery bypass surgery when it is administrated to maintain anesthesia at 1 minimum alveolar concentration (MAC), shown by improved clinical results and myocardial injury biomarkers after surgery (60, 61). A recent meta-analysis reports that halogenated agents including desflurane, isoflurane, and sevoflurane can reduce the occurrence of myocardial infarction, mortality rate, and mechanical ventilation need after cardiac surgery, especially for the patients undergoing CABG surgery (62). However, a meta-analysis of 79 randomized controlled trials (RCTs) including 6,219 patients with noncardiac surgery reports that cardioprotective benefits of sevoflurane are inconclusive (63). It should be noted that this meta-analysis uses perioperative myocardial infarction or deaths as the main endpoint and no case occurs in any of the included studies. Similarly, another systematic review and meta-analysis indicate that the use of volatile anesthetics such as sevoflurane, desflurane, or isoflurane is associated with decreased mortality and perioperative complications in patients undergoing cardiac surgery, but does not improve these outcomes in patients with noncardiac surgery (64). It is still unclear why cardioprotection of sevoflurane is significantly different between patients with cardiac and noncardiac surgeries. Thus, further studies are needed to determine whether cardioprotection of sevoflurane is really surgery type-dependent and which factors can impair the transformation of cardioprotective benefits provided by sevoflurane into improved clinical outcomes of patients.

Tracing the potential sources responsible for heterogeneity in a meta-analysis can guide preclinical and clinical study designs. As our analysis showed that there were high levels of heterogeneity between the included studies, both the meta-regression and stratified analysis were used to explore the potential sources of heterogeneity and determine whether some of the predefined experimental variables in this analysis could influence the observed effect size with cardioprotective efficacy of sevoflurane conditionings. In fact, this method for analyzing heterogeneity has been successfully applied to several promising preclinical interventions (65, 66). Our stratified analysis showed that animal species (dog, rabbit, rat, and mice) did not produce significant effects on either effect size or heterogeneity of both the SPreC and SPostC groups. In addition, meta-regression suggested that country difference might be a source of significant heterogeneity for SPreC. Further stratified analysis by countries showed that there was still significant heterogeneity between the groups. Compared to other countries, moreover, Netherlands showed a large WMD, with a wide 95% CI. This may partly explain the source of heterogeneity. Anyway, it should be aware that preclinical characterization of sevoflurane administration protocol *in-vivo* studies has not yet been identified and current studies have showed uneven quality. All of these are essential to determine the important parameters that will facilitate optimization of administration protocol in further clinical trials.

Certainly, both the poor methodological quality and publication bias can result in over- or under-estimation of effect size (67, 68). To determine the internal validity of included studies in this analysis, both the quality of study and publication bias were evaluated by the reporting quality assessment tools, i.e., the ARRIVE guidelines 2.0 and a 12-item quality score. Our results showed that the overall scores of the reporting quality assessment tools for the included studies were high, indicating the validity of this analysis. In fact, main contents of experimental process, such as confirmation of ischemia and monitoring of ECG, were well reported in the included studies. However, a number of aspects required by the ARRIVE guidelines 2.0 were poorly reported in the most included studies, particularly in sample size calculations (11%) and blinding (16%). These may inevitably raise concerns of statistical validity and result in exclusion of literature selection. Furthermore, appropriate monitoring of experimental animals, such as PaO_2_ or SaO_2_ (27%), depth of anesthesia (22%), and concentration of sevoflurane (38%), was also reported poorly in the included studies. According to a position article on improving the preclinical assessment of novel cardioprotective therapies (69), these factors are actually essential to ensure a high-quality study. Thus, failure to report high-quality studies may partly account for the observed heterogeneity of this analysis. Nevertheless, the effect sizes by sevoflurane conditionings were consistent and robust despite high heterogeneity was observed.

Finally, publication bias within the included studies was visually assessed by a funnel plot and further detected by the Egger's and Begg's tests. Notably, publication bias was displayed in the funnel plot and identified by Egger's test (*P* < 0.01) for the SPostC group. Visual analysis of the funnel plot suggested that neutral or negative studies might be underrepresented in the SPostC group. That is true, studies with neutral or negative data are often not given priority for publication. Fortunately, it is reassuring that the bias of our analysis does not result in a statistically significant impact on the overall effect.

## Limitations

This meta-analysis has included all the studies, which met our rigorous inclusion criteria. Besides, the studies that are not published and do not meet important quality criteria are not be included in our analysis. As the validity of a meta-analysis is highly depended on the quality of all the included studies, there are several limitations in this analysis that deserve special attention. First, there is only a small amount of data from large animals in our analysis, though large animals share more anatomical and physiological characteristics of heart with human. This may limit the interpretation and extension of our results. It is warranted that large animal experiments are needed to further confirm the favorable effects of sevoflurane conditionings on myocardial IRI in small animal models. Second, as mentioned in the discussion, this analysis only includes the studies that are conducted on normal animals without any risk factors of cardiovascular diseases, such as aging, diabetes, obesity, hyperlipidemia, hypertension, and others. Evidently, our results cannot be generalized to the animals with risk factors of cardiovascular diseases. Third, besides country differences may be a source of heterogeneity for SPreC studies, meta-regression fails to reveal any influence of other prespecified covariates on pooled estimates of infarct size. However, robustness of the data is evidenced by both the sensitivity analysis and stratified analysis, which confirm the benefits and reliability of sevoflurane conditionings in ameliorating myocardial IRI. Thus, the real reasons for high degree of heterogeneity among the included studies are unclear. To address above issues and confirm therapeutic effect of sevoflurane conditionings on myocardial IRI, we believe that more animal studies and RCTs are still required.

## Conclusion

This comprehensive systematic review and meta-analysis demonstrate that sevoflurane conditionings can provide a robust and highly reproducible infarct size-sparing effect in animal models of myocardial IRI. Furthermore, this beneficial effect can be obtained when sevoflurane conditioning is carried out before the onset of ischemia, during ischemia, or during reperfusion, despite a high heterogeneity among the included studies is observed. Nonetheless, preclinical studies with a high-quality design, especially for those conducted on large animal models and the animals with risk factors of cardiovascular diseases, are still required to future determine the protection of sevoflurane conditionings against myocardial IRI and explore relevant mechanisms.

## Data Availability Statement

The original contributions presented in the study are included in the article/Supplementary Material, further inquiries can be directed to the corresponding author.

## Author Contributions

BH and F-SX contributed to the study design, planning, data analysis, and wrote the manuscript. TT, P-PH, W-CL, Y-GC, and T-YJ contributed to data analysis and revision of the manuscript. All authors had seen and approved the final version of the manuscript.

## Funding

This study was performed with the financial support of the National Natural Science Foundation of China (No. 81470019 to F-SX).

## Conflict of Interest

The authors declare that the research was conducted in the absence of any commercial or financial relationships that could be construed as a potential conflict of interest.

## Publisher's Note

All claims expressed in this article are solely those of the authors and do not necessarily represent those of their affiliated organizations, or those of the publisher, the editors and the reviewers. Any product that may be evaluated in this article, or claim that may be made by its manufacturer, is not guaranteed or endorsed by the publisher.

## Supplementary Material

Supplementary Table 1 Sensitivity analysis.

Supplementary Table 2 The proposed mechanism of cardioprotection by sevoflurane preconditioning (SPreC) and sevoflurane postconditioning (SPostC) in the included studies.

Supplementary Material 1 **(A)** Web of Science search history; **(B)** Cochrane search history; **(C)** Embase search history; and **(D)** PubMed search history.

Supplementary Material 2 Meta-regression.

Supplementary Material 3 Stratified analysis.
