# Supplementary material for: The Protective Effect of Sevoflurane Conditionings Against Myocardial Ischemia/Reperfusion Injury: A Systematic Review and Meta-Analysis of Preclinical Trials in in-vivo Models
Source: Front Cardiovasc Med. 2022 Apr 28;9:841654. doi: 10.3389/fcvm.2022.841654 (PMC9095933; doi:10.3389/fcvm.2022.841654)
Supplement: Supplementary Table 1 — Sensitivity analysis. [file Table_1.DOCX]

**Supplementary table 1** Sensitivity analysis

| **Studies omitted** | **WMD** | **95%CI** | ***P* values** | **Heterogeneity** |
| --- | --- | --- | --- | --- |
| **SPreC** | | | | |
| Toller 1999  Toller 1999  Obal 2005 | -18.89  -19.16  -18.32 | -23.76, -14.02  -23.92, -14.39  -23.12, -13.52 | *P* < 0.01  *P* < 0.01  *P* < 0.01 | *I^2^* = 94%, *P* < 0.01  *I^2^* = 94%, *P* < 0.01  *I^2^* = 94%, *P* < 0.01 |
| Lange 2006 | -18.35 | -23.16, -13.54 | *P* < 0.01 | *I^2^* = 94%, *P* < 0.01 |
| Redel 2009 | -18.45 | -23.23, -13.66 | *P* < 0.01 | *I^2^* = 94%, *P* < 0.01 |
| Wang 2010 | -18.54 | -23.43, -13.64 | *P* < 0.01 | *I^2^* = 94%, *P* < 0.01 |
| Frassdorf 2010 | -18.31 | -23.17, -13.45 | *P* < 0.01 | *I^2^* = 94%, *P* < 0.01 |
| Frassdorf 2010 | -17.95 | -22.70, -13.21 | *P* < 0.01 | *I^2^* = 94%, *P* < 0.01 |
| Frassdorf 2010 | -17.12 | -21.08, -13.17 | *P* < 0.01 | *I^2^* = 91%, *P* < 0.01 |
| Frassdorf 2010 | -17.30 | -21.36, -13.23 | *P* < 0.01 | *I^2^* = 92%, *P* < 0.01 |
| Tosaka 2011 | -18.28 | -23.12, -13.45 | *P* < 0.01 | *I^2^* = 94%, *P* < 0.01 |
| Xiao 2011 | -18.54 | -23.39, -13.69 | *P* < 0.01 | *I^2^* = 94%, *P* < 0.01 |
| Zhang 2012 | -18.65 | -23.60, -13.71 | *P* < 0.01 | *I^2^* = 94%, *P* < 0.01 |
| Ma 2013 | -15.58 | -23.51, -13.64 | *P* < 0.01 | *I^2^* = 94%, *P* < 0.01 |
| Qiao 2013 | -18.61 | -23.50, -13.73 | *P* < 0.01 | *I^2^* = 94%, *P* < 0.01 |
| Zhao 2013 | -19.01 | -23.83, -14.20 | *P* < 0.01 | *I^2^* = 94%, *P* < 0.01 |
| Xie 2014 | -18.58 | -23.49, -13.66 | *P* < 0.01 | *I^2^* = 94%, *P* < 0.01 |
| Behmenburg 2017  Behmenburg 2017  Behmenburg 2017  Behmenburg 2017  Behmenburg 2017  Behmenburg 2017  Behmenburg 2017  Behmenburg 2017  Liu 2019 | -18.76  -18.87  -18.83  -19.32  -18.72  -18.65  -18.72  -19.19  -18.65 | -23.61, -13.92  -23.69, -14.04  -23.66, -14.00  -24.03, -14.61  -23.57, -13.88  -23.49, -13.81  -23.55, -13.89  -23.95, -14.42  -23.60, -13.70 | *P* < 0.01  *P* < 0.01  *P* < 0.01  *P* < 0.01  *P* < 0.01  *P* < 0.01  *P* < 0.01  *P* < 0.01  *P* < 0.01 | *I^2^* = 94%, *P* < 0.01  *I^2^* = 94%, *P* < 0.01  *I^2^* = 94%, *P* < 0.01  *I^2^* = 94%, *P* < 0.01  *I^2^* = 94%, *P* < 0.01  *I^2^* = 94%, *P* < 0.01  *I^2^* = 94%, *P* < 0.01  *I^2^* = 94%, *P* < 0.01  *I^2^* = 94%, *P* < 0.01 |
| Xie 2020  Hong 2020 | -18.69  -18.54 | -23.95, -13.43  -23.46, -13.62 | *P* < 0.01  *P* < 0.01 | *I^2^* = 94%, *P* < 0.01  *I^2^* = 94%, *P* < 0.01 |
| Combined | -18.56 | -23.27, -13.85 | *P* < 0.01 | *I^2^* = 94%, *P* < 0.01 |
| **SPostC** | | | | |
| Preckel 1998 | -18.48 | -21.04, -15.91 | *P* < 0.01 | *I^2^* = 91%, *P* < 0.01 |
| Obal 2001 | -18.62 | -21.18, -16.07 | *P* < 0.01 | *I^2^* = 91%, *P* < 0.01 |
| Obal 2001 | -18.42 | -20.98, -15.86 | *P* < 0.01 | *I^2^* = 91%, *P* < 0.01 |
| Obal 2001 | -18.42 | -20.98, -15.86 | *P* < 0.01 | *I^2^* = 91%, *P* < 0.01 |
| Obal 2001 | -18.41 | -20.97, -15.86 | *P* < 0.01 | *I^2^* = 91%, *P* < 0.01 |
| Obal 2003 | -17.88 | -20.36, -15.40 | *P* < 0.01 | *I^2^* = 90%, *P* < 0.01 |
| Obal 2003 | -18.19 | -20.74, -15.64 | *P* < 0.01 | *I^2^* = 91%, *P* < 0.01 |
| Obal 2003 | -18.19 | -20.74, -15.64 | *P* < 0.01 | *I^2^* = 91%, *P* < 0.01 |
| Obal 2005  Huhn 2008  Redel 2009  Tosaka 2011  Drenger 2011 | -18.07  -18.36  -18.09  -18.17  -18.69 | -20.60, -15.53  -20.93, -15.80  -20.62, -15.56  -20.72, -15.62  -21.33, -16.04 | *P* < 0.01  *P* < 0.01  *P* < 0.01  *P* < 0.01  *P* < 0.01 | *I^2^* = 90%, *P* < 0.01  *I^2^* = 91%, *P* < 0.01  *I^2^* = 91%, *P* < 0.01  *I^2^* = 90%, *P* < 0.01  *I^2^* = 87%, *P* < 0.01 |
| Tai 2012  Chen 2012  Chen 2012  Chen 2012  Chen 2012  Chen 2012  Chen 2012  Chen 2012  Chen 2012  Chen 2012  Xu 2013  Li 2013  Zhang 2014 | -18.36  -18.80  -17.95  -18.47  -18.60  -18.21  -18.16  -18.55  -18.52  -18.48  -18.40  -18.24  -18.20 | -20.94, -15.79  -21.35, -16.25  -20.51, -15.39  -21.04, -15.90  -21.15, -16.06  -20.76, -15.67  -20.70, -15.62  -21.10, -15.99  -21.08, -15.97  -21.03, -15.92  -20.98, -15.81  -20.79, -15.68  -20.75, -15.66 | *P* < 0.01  *P* < 0.01  *P* < 0.01  *P* < 0.01  *P* < 0.01  *P* < 0.01  *P* < 0.01  *P* < 0.01  *P* < 0.01  *P* < 0.01  *P* <0.01  *P* < 0.01  *P* < 0.01 | *I^2^* = 91%, *P* < 0.01  *I^2^* = 91%, *P* < 0.01  *I^2^* = 91%, *P* < 0.01  *I^2^* = 91%, *P* < 0.01  *I^2^* = 91%, *P* < 0.01  *I^2^* = 91%, *P* < 0.01  *I^2^* = 91%, *P* < 0.01  *I^2^* = 91%, *P* < 0.01  *I^2^* = 91%, *P* < 0.01  *I^2^* = 91%, *P* < 0.01  *I^2^* = 91%, *P* < 0.01  *I^2^* = 90%, *P* < 0.01  *I^2^* = 90%, *P* < 0.01 |
| Stumpner 2014  Gao 2016  Lin 2016 | -18.11  -18.60  -18.54 | -20.65, -15.58  -21.18, -16.02  -21.11, -15.97 | *P* < 0.01  *P* < 0.01  *P* < 0.01 | *I^2^* = 90%, *P* < 0.01  *I^2^* = 91%, *P* < 0.01  *I^2^* = 91%, *P* < 0.01 |
| Li 2016 | -18.19 | -20.74, -15.64 | *P* < 0.01 | *I^2^* = 90%, *P* < 0.01 |
| Li 2016  Qiao 2019 | -18.12  -18.40 | -20.66, -15.59  -20.98, -15.81 | *P* < 0.01  *P* < 0.01 | *I^2^* = 90%, *P* < 0.01  *I^2^* = 91%, *P* < 0.01 |
| Qi 2019 | -18.40 | -21.00, -15.80 | *P* < 0.01 | *I^2^* = 91%, *P* < 0.01 |
| Qi 2019 | -17.85 | -20.14, -15.57 | *P* < 0.01 | *I^2^* = 87%, *P* < 0.01 |
| Huang 2019 | -18.54 | -21.25, -15.83 | *P* < 0.01 | *I^2^* = 91%, *P* < 0.01 |
| Tan 2020 | -18.46 | -21.03, -15.89 | *P* < 0.01 | *I^2^* = 91%, *P* < 0.01 |
| Tan 2020 | -18.21 | -20.77, -15.66 | *P* < 0.01 | *I^2^* = 91%, *P* < 0.01 |
| Tan 2020  Yu 2021  Gao 2021 | -18.00  -18.60  -18.64 | -20.52, -15.49  -21.41, -15.80  -21.31, -15.97 | *P* < 0.01  *P* < 0.01  *P* < 0.01 | *I^2^* = 90%, *P* < 0.01  *I^2^* = 91%, *P* < 0.01  *I^2^* = 91%, *P* < 0.01 |
| Combined | -18.35 | -20.88, -15.83 | *P* < 0.01 | *I^2^* = 90%, *P* < 0.01 |

**Note:** For study listed in the first column, if its last name of first author and year of publication are repeated, it indicates that same study involves different intervention protocols. Please refer to the above Table 1 for details. Please refer to the above Table 1 for details of references.
