# Supplementary material for: The Protective Effect of Sevoflurane Conditionings Against Myocardial Ischemia/Reperfusion Injury: A Systematic Review and Meta-Analysis of Preclinical Trials in in-vivo Models
Source: Front Cardiovasc Med. 2022 Apr 28;9:841654. doi: 10.3389/fcvm.2022.841654 (PMC9095933; doi:10.3389/fcvm.2022.841654)
Supplement: Supplementary Table 2 — The proposed mechanism of cardioprotection by sevoflurane preconditioning (SPreC) and sevoflurane postconditioning (SPostC) in the included studies. [file Table_2.DOCX]

**Supplementary table 2** The proposed mechanism of cardioprotection by SPreC and SPostC in the included studies

| **Studies ID** | | **Proposed mechanisms** |
| --- | --- | --- |
| **SPreC** | | |
| **Toller 1999** | | Activating K_ATP_ channels |
| **Obal 2005** | | Opening mK_ATP_-channels |
| **Lange 2006** | | Activating β1-adrenergic pathway |
| **Redel 2009** | | NA |
| **Wang 2010** | | NF-κB upregulates Bcl-2 in the period of sevoflurane preconditioning and downregulates inflammatory proteins of ICAM-1 and TNF-α in the period of reperfusion |
| **Frassdorf 2010** | | Inducing endothelial NOS phosphorylation |
| **Tosaka 2011** | | Mediating cardioprotection via both COX-2-dependent and -independent pathways |
| **Xiao 2011** | | Inducing mitochondrial proteome remodeling, which mainly involves proteins that are related to ATP generation and transport |
| **Zhang 2012** | | Upregulating expression of iNOS and activating of downstream mK_ATP_ |
| **Ma 2013** | | Upregulating expression of myocardial p-Akt and p-ERK1/2 |
| **Qiao 2013** | | Activating the transcription factor NF-κB and upregulating autophagy, reducing inflammation (TNF-α, IL-1β) and apoptosis (caspase-3) |
| **Zhao 2013** | | Caveolin-3-dependent cyclooxygenase-2 inhibition and antioxidative effects (LDH, SOD) |
| **Xie 2014** | | Anti-apoptosis (TUNEL assay and caspase-3) |
| **Behmenburg 2017** | | NA |
| **Liu 2019** | | Attenuating apoptosis and preventing ERS activation by suppressing PERK/eIF2α/ATF4/CHOP signaling via activating the Akt pathway |
| **Xie 2020** | | AMPK-dependent inhibition of pro-death MAPK |
| **Hong 2020** | | Activating of AMPK and restoring of I/R-impaired autophagic flux (LC3II/I ratios, Beclin-1 and P62) |
| **SPostC** | | |
| **Preckel 1998** | NA | |
| **Obal 2001** | NA | |
| **Obal 2003** | Blocking calcium release from sarcoplasmic reticulum (possible mechanism) | |
| **Huhn 2008** | Inhibiting the opening of mPTP | |
| **Drenger 2011** | Activating PI3K pathway and mK_ATP_ | |
| **Tai 2012** | Enhancing phosphorylation of GSK-3β Ser^9^, Akt Ser^473^ and ERK1/2 in NDRs | |
| **Chen 2012** | NA | |
| **Xu 2013** | Increasing phosphorylation of Akt, ERK1/2 and GSK3β | |
| **Li 2013** | Activating Akt and ERK1/2 to inhibit mPTP opening | |
| **Zhang 2014** | Restoring autophagic flux: declining in autophagosome accumulation, attenuating autophagic markers, and improving lysosomal function | |
| **Stumpner 2014** | Activating COX-2 | |
| **Gao 2016** | Activating Nrf2/Brg1/HO-1 signaling | |
| **Lin 2016** | Increasing expression of p-STAT3, APN restoration, and decreasing expression of Fox1 and CD36 | |
| **Li 2016** | Activating Akt and ERK1/2 and anti-apoptosis (apoptotic index) | |
| **Zhang 2018** | Decreasing LINC00652 expression, increasing GLP-1R expression, decreasing myocardial cell apoptosis, L-1β and TNF-α level | |
| **Qiao 2019** | Resulting in higher eNOS and nNOS phosphorylation levels, NOS content, and NO production, decreasing mPTP opening and cytochrome c release and restoring I/R-impaired autophagic flux | |
| **Qi 2019** | Upregulating miR-145 expression and downregulating GZMK expression | |
| **Huang 2019** | Downregulating miR-155 expression to promote the expression of SIRT1, reducing pathological injuries and reducing cardiomyocyte apoptosis | |
| **Tan 2020** | Upregulating miR-203 to reduce DCX expression, thereby repressing oxidative stress (GSH, SOD, MDA), inflammation (TNF-α, IL-6, IL-1α), apoptosis (Cleaved caspase-3, apoptotic index) | |
| **Yu 2021** | Inhibiting mitochondrial fission | |
| **Gao 2021** | Through TOPK-mediated PTEN/PI3K/Akt activation | |

**Note:** For study listed in the first column, if its last name of first author and year of publication are repeated, it indicates that same study involves different intervention protocols. Please refer to the above Table 1 for details. Please refer to the above Table 1 for details of references.
