# Supplementary material for: The Protective Effect of Sevoflurane Conditionings Against Myocardial Ischemia/Reperfusion Injury: A Systematic Review and Meta-Analysis of Preclinical Trials in in-vivo Models
Source: Front Cardiovasc Med. 2022 Apr 28;9:841654. doi: 10.3389/fcvm.2022.841654 (PMC9095933; doi:10.3389/fcvm.2022.841654)
Supplement: Supplementary Material 1 — (A) Web of Science search history; (B) Cochrane search history; (C) Embase search history; and (D) PubMed search history. [file Table_3.DOCX]

**A: Web of science SearchHistory**

|  | | | |  |
| --- | --- | --- | --- | --- |
| # 3 | [**495**](https://apps.webofknowledge.com/summary.do?product=WOS&doc=1&qid=3&SID=5EvwkkkZu4SmLIsAIj8&search_mode=CombineSearches&update_back2search_link_param=yes) | #2 AND #1  *INDEXS=SCI-EXPANDED, SSCI, A&HCI, CPCI-S, CPCI-SSH, ESCI, CCR-EXPANDED, IC TIME INTERVAL= ALL YEARS* |  | |
|  | | | |  |
| # 2 | [**133,633**](https://apps.webofknowledge.com/summary.do?product=WOS&doc=1&qid=2&SID=5EvwkkkZu4SmLIsAIj8&search_mode=AdvancedSearch&update_back2search_link_param=yes) | TS=(Myocardial Reperfusion Injury OR Injuries, Myocardial Reperfusion OR Myocardial Reperfusion Injuries OR Reperfusion Injuries, Myocardial OR Myocardial Ischemic Reperfusion Injury OR Reperfusion Injury, Myocardial OR Injury, Myocardial Reperfusion OR Myocardial Ischemia OR Ischemia, Myocardial OR Ischemias, Myocardial OR Myocardial Ischemias OR Ischemic Heart Disease OR Heart Disease, Ischemic OR Disease, Ischemic Heart OR Diseases, Ischemic Heart OR Heart Diseases, Ischemic OR Ischemic Heart Diseases OR Myocardial Reperfusion OR Reperfusion, Myocardial OR Myocardial Reperfusions OR Reperfusions, Myocardial OR Coronary Reperfusion OR Coronary Reperfusions OR Reperfusion, Coronary OR Reperfusions, Coronary OR Myocardial ischemia/Reperfusion injury OR Myocardial ischemia-Reperfusion injury OR Myocardial IR injury)  *INDEXS =SCI-EXPANDED, SSCI, A&HCI, CPCI-S, CPCI-SSH, ESCI, CCR-EXPANDED, IC TIME INTERVAL= ALL YEARS* |  | |
|  | | | |  |
| # 1 | [**12,417**](https://apps.webofknowledge.com/summary.do?product=WOS&doc=1&qid=1&SID=5EvwkkkZu4SmLIsAIj8&search_mode=AdvancedSearch&update_back2search_link_param=yes) | TS= (Sevoflurane OR Fluoromethyl-2,2,2-trifluoro-1-(trifluoromethyl) ethyl Ether OR Fluoromethyl Hexafluoroisopropyl Ether OR Sevorane OR Ultane OR BAX 3084)  *INDEXS =SCI-EXPANDED, SSCI, A&HCI, CPCI-S, CPCI-SSH, ESCI, CCR-EXPANDED, IC TIME INTERVAL= ALL YEARS* |  | |
|  | | | |  |

**B: Cochrane Search History**

Search Name: Sevofluranemeta

Last Saved: 28/07/2021 22:51:57

Comment: Cochranesearchhistory

ID Search

#1 MeSH descriptor: [Sevoflurane] explode all trees

#2 MeSH descriptor: [Myocardial Reperfusion Injury] explode all trees

#3 MeSH descriptor: [Myocardial Ischemia] explode all trees

#4 MeSH descriptor: [Myocardial Reperfusion] explode all trees

#5 (Fluoromethyl Hexafluoroisopropyl Ether):ti,ab,kw OR (Sevorane):ti,ab,kw OR (Ultane):ti,ab,kw OR (BAX 3084):ti,ab,kw

#6 (Injuries, Myocardial Reperfusion):ti,ab,kw OR (Myocardial Reperfusion Injuries):ti,ab,kw OR (Reperfusion Injuries, Myocardial):ti,ab,kw OR (Myocardial Ischemic Reperfusion Injury):ti,ab,kw OR (Reperfusion Injury, Myocardial):ti,ab,kw OR (Injury, Myocardial Reperfusion):ti,ab,kw OR (Ischemia, Myocardial):ti,ab,kw OR (Ischemias, Myocardial):ti,ab,kw OR (Myocardial Ischemias):ti,ab,kw OR (Ischemic Heart Disease):ti,ab,kw OR (Heart Disease, Ischemic):ti,ab,kw OR (Disease, Ischemic Heart):ti,ab,kw OR (Diseases, Ischemic Heart):ti,ab,kw OR (Heart Diseases, Ischemic):ti,ab,kw OR (Ischemic Heart Diseases):ti,ab,kw OR (Reperfusion, Myocardial ):ti,ab,kw OR (Myocardial Reperfusions):ti,ab,kw OR (Reperfusions, Myocardial):ti,ab,kw OR (Coronary Reperfusion):ti,ab,kw OR (Coronary Reperfusions):ti,ab,kw OR (Reperfusion, Coronary):ti,ab,kw OR (Reperfusions, Coronary):ti,ab,kw

#7 #1 OR #5

#8 #2 OR #3 OR #4 OR #6

#9 #7 AND #8

**C: Embase Search History**

| **No.** | **Query** | **Results** | **Date** | |  |
| --- | --- | --- | --- | --- | --- |
| 7# | #5 AND #6 | 301 | 2021/7/29 | |  |
| 6# | #2 OR #4 | 82693 | 2021/7/29 | |  |
| 5# | #1 OR #3 | 22237 | 2021/7/29 | |  |
| 4# | 'myocardial reperfusion injury':ab,ti OR 'injuries, myocardial reperfusion':ab,ti OR 'myocardial reperfusion injuries':ab,ti OR 'reperfusion injuries, myocardial':ab,ti OR 'myocardial ischemic reperfusion injury':ab,ti OR 'reperfusion injury, myocardial':ab,ti OR 'injury, myocardial reperfusion':ab,ti OR 'myocardial ischemia':ab,ti OR 'ischemia, myocardial':ab,ti OR 'ischemias, myocardial':ab,ti OR 'myocardial ischemias':ab,ti OR 'ischemic heart disease':ab,ti OR 'heart disease, ischemic':ab,ti OR 'disease, ischemic heart':ab,ti OR 'diseases, ischemic heart':ab,ti OR 'heart diseases, ischemic':ab,ti OR 'ischemic heart diseases':ab,ti OR 'myocardial reperfusion':ab,ti OR 'reperfusion, myocardial':ab,ti OR 'myocardial reperfusions':ab,ti OR 'reperfusions, myocardial':ab,ti OR 'coronary reperfusion':ab,ti OR 'coronary reperfusions':ab,ti OR 'reperfusion, coronary':ab,ti OR 'reperfusions, coronary':ab,ti | 80490 | 2021/7/29 | |  |
| 3# | 'fluoromethyl-2,2,2-trifluoro-1-(trifluoromethyl)ethyl ether':ab,ti OR 'fluoromethyl hexafluoroisopropyl ether':ab,ti OR 'sevorane':ab,ti OR 'ultane':ab,ti OR 'bax 3084':ab,ti | 62 | 2021/7/29 | |  |
| 2# | 'myocardial ischemia reperfusion injury'/exp | 4513 | 2021/7/29 | |  |
| 1# | 'sevoflurane'/exp | 22228 | | 2021/7/29 | |

**D: PubMed Search History**

**Search number**

1

**Query**

((((((Fluoromethyl-2,2,2-trifluoro-1-(trifluoromethyl)ethyl Ether[Title/Abstract]) OR (Fluoromethyl Hexafluoroisopropyl Ether[Title/Abstract])) OR (Sevorane[Title/Abstract])) OR (Ultane[Title/Abstract])) OR (BAX 3084[Title/Abstract])) OR ("Sevoflurane"[Mesh])) AND ((((("Myocardial Reperfusion"[Mesh]) OR (((((((Reperfusion, Myocardial[Title/Abstract]) OR (Myocardial Reperfusions[Title/Abstract])) OR (Reperfusions, Myocardial[Title/Abstract])) OR (Coronary Reperfusion[Title/Abstract])) OR (Coronary Reperfusions[Title/Abstract])) OR (Reperfusion, Coronary[Title/Abstract])) OR (Reperfusions, Coronary[Title/Abstract]))) OR (("Myocardial Ischemia"[Mesh]) OR (((((((((Ischemia, Myocardial[Title/Abstract]) OR (Ischemias, Myocardial[Title/Abstract])) OR (Myocardial Ischemias[Title/Abstract])) OR (Ischemic Heart Disease[Title/Abstract])) OR (Heart Disease, Ischemic[Title/Abstract])) OR (Disease, Ischemic Heart[Title/Abstract])) OR (Diseases, Ischemic Heart[Title/Abstract])) OR (Heart Diseases, Ischemic[Title/Abstract])) OR (Ischemic Heart Diseases[Title/Abstract])))) OR (("Myocardial Reperfusion Injury"[Mesh]) OR ((((((Injuries, Myocardial Reperfusion[Title/Abstract]) OR (Myocardial Reperfusion Injuries[Title/Abstract])) OR (Reperfusion Injuries, Myocardial[Title/Abstract])) OR (Myocardial Ischemic Reperfusion Injury[Title/Abstract])) OR (Reperfusion Injury, Myocardial[Title/Abstract])) OR (Injury, Myocardial Reperfusion[Title/Abstract])))))

**Sort By**

Publication Date

**Filters**

-

**Search Details**

(((("Fluoromethyl-2"[All Fields] AND "2"[All Fields] AND "2 trifluoro 1"[All Fields]) AND ("trifluoromethyl"[All Fields] OR "trifluoromethylated"[All Fields] OR "trifluoromethylating"[All Fields] OR "trifluoromethylation"[All Fields] OR "trifluoromethylations"[All Fields] OR "trifluoromethylative"[All Fields])) AND "ethyl ether"[Title/Abstract]) OR (("fluoromethyl"[All Fields] OR "fluoromethylated"[All Fields] OR "fluoromethylation"[All Fields]) AND "hexafluoroisopropyl ether"[Title/Abstract]) OR "Sevorane"[Title/Abstract] OR "Ultane"[Title/Abstract] OR "bax 3084"[Title/Abstract] OR "Sevoflurane"[MeSH Terms]) AND ("Myocardial Reperfusion"[MeSH Terms] OR ("reperfusion myocardial"[Title/Abstract] OR (("myocardially"[All Fields] OR "myocardium"[MeSH Terms] OR "myocardium"[All Fields] OR "Myocardial"[All Fields]) AND "Reperfusions"[Title/Abstract]) OR (("reperfusate"[All Fields] OR "reperfusates"[All Fields] OR "reperfuse"[All Fields] OR "reperfused"[All Fields] OR "reperfusing"[All Fields] OR "Reperfusion"[MeSH Terms] OR "Reperfusion"[All Fields] OR "Reperfusions"[All Fields] OR "reperfusive"[All Fields]) AND "Myocardial"[Title/Abstract]) OR "coronary reperfusion"[Title/Abstract] OR (("coronaries"[All Fields] OR "Heart"[MeSH Terms] OR "Heart"[All Fields] OR "Coronary"[All Fields]) AND "Reperfusions"[Title/Abstract]) OR "reperfusion coronary"[Title/Abstract] OR (("reperfusate"[All Fields] OR "reperfusates"[All Fields] OR "reperfuse"[All Fields] OR "reperfused"[All Fields] OR "reperfusing"[All Fields] OR "Reperfusion"[MeSH Terms] OR "Reperfusion"[All Fields] OR "Reperfusions"[All Fields] OR "reperfusive"[All Fields]) AND "Coronary"[Title/Abstract])) OR ("Myocardial Ischemia"[MeSH Terms] OR ("ischemia myocardial"[Title/Abstract] OR "ischemias myocardial"[Title/Abstract] OR "myocardial ischemias"[Title/Abstract] OR "ischemic heart disease"[Title/Abstract] OR "heart disease ischemic"[Title/Abstract] OR "disease ischemic heart"[Title/Abstract] OR "diseases ischemic heart"[Title/Abstract] OR "heart diseases ischemic"[Title/Abstract] OR "ischemic heart diseases"[Title/Abstract])) OR ("Myocardial Reperfusion Injury"[MeSH Terms] OR ((("injurie"[All Fields] OR "injuried"[All Fields] OR "Injuries"[MeSH Subheading] OR "Injuries"[All Fields] OR "wounds and injuries"[MeSH Terms] OR ("wounds"[All Fields] AND "Injuries"[All Fields]) OR "wounds and injuries"[All Fields] OR "injurious"[All Fields] OR "injury s"[All Fields] OR "injuryed"[All Fields] OR "injurys"[All Fields] OR "Injury"[All Fields]) AND "Myocardial Reperfusion"[Title/Abstract]) OR "myocardial reperfusion injuries"[Title/Abstract] OR "reperfusion injuries myocardial"[Title/Abstract] OR "myocardial ischemic reperfusion injury"[Title/Abstract] OR "reperfusion injury myocardial"[Title/Abstract] OR "injury myocardial reperfusion"[Title/Abstract])))

**Results**

282

**Time**

10:17:23
