# Supplementary material for: The Protective Effect of Sevoflurane Conditionings Against Myocardial Ischemia/Reperfusion Injury: A Systematic Review and Meta-Analysis of Preclinical Trials in in-vivo Models
Source: Front Cardiovasc Med. 2022 Apr 28;9:841654. doi: 10.3389/fcvm.2022.841654 (PMC9095933; doi:10.3389/fcvm.2022.841654)
Supplement: Supplementary Material 2 — Meta-regression. [file Table_4.DOCX]

**Note：**Meta-regression of SPreC group.

**Note：**Meta-regression of SPostC group.
