# Supplementary material for: The Protective Effect of Sevoflurane Conditionings Against Myocardial Ischemia/Reperfusion Injury: A Systematic Review and Meta-Analysis of Preclinical Trials in in-vivo Models
Source: Front Cardiovasc Med. 2022 Apr 28;9:841654. doi: 10.3389/fcvm.2022.841654 (PMC9095933; doi:10.3389/fcvm.2022.841654)
Supplement: Supplementary Material 3 — Stratified analysis. [file Table_5.DOCX]

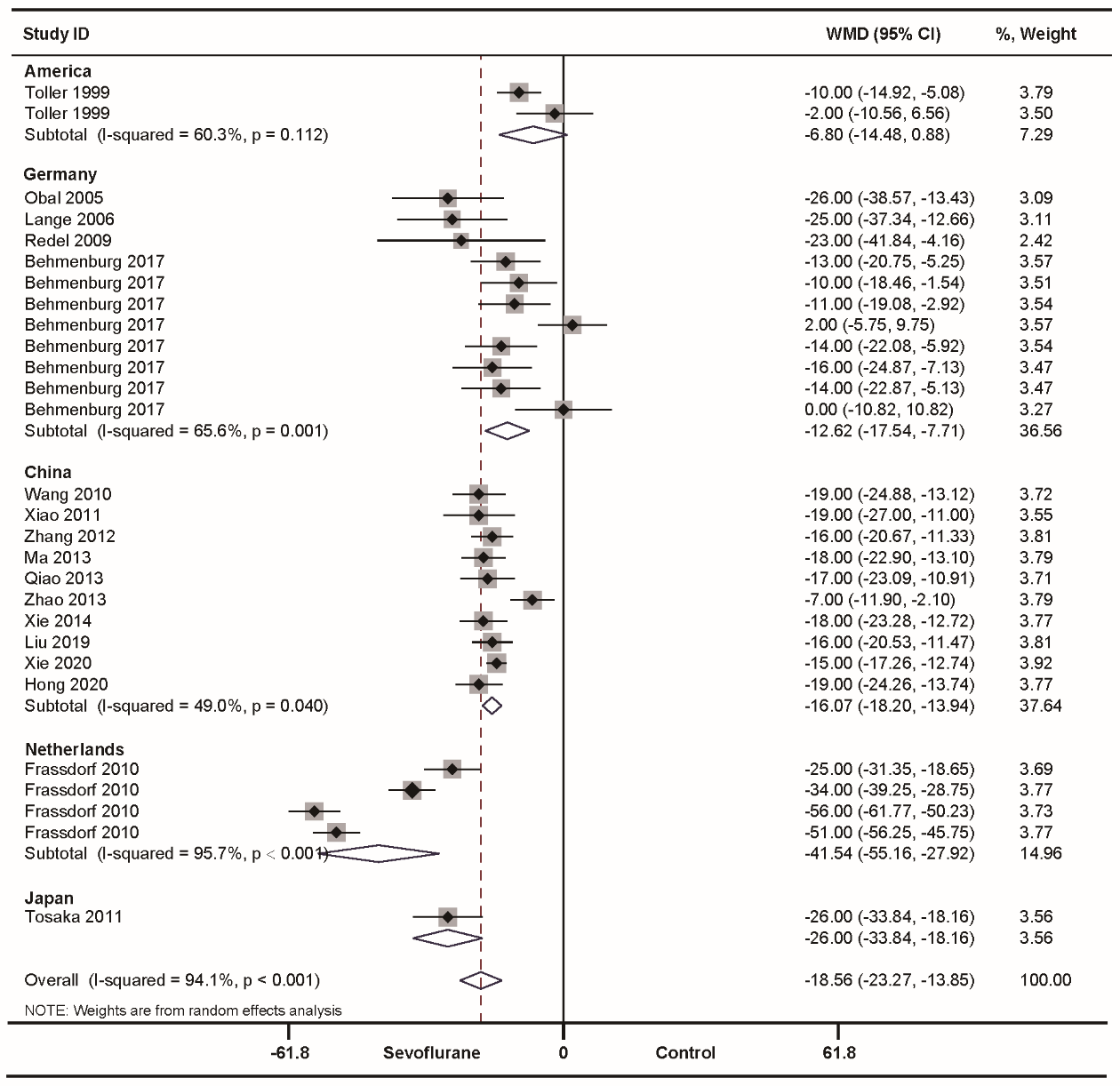
**Note：**Stratified analysis by countries in SPreC group.


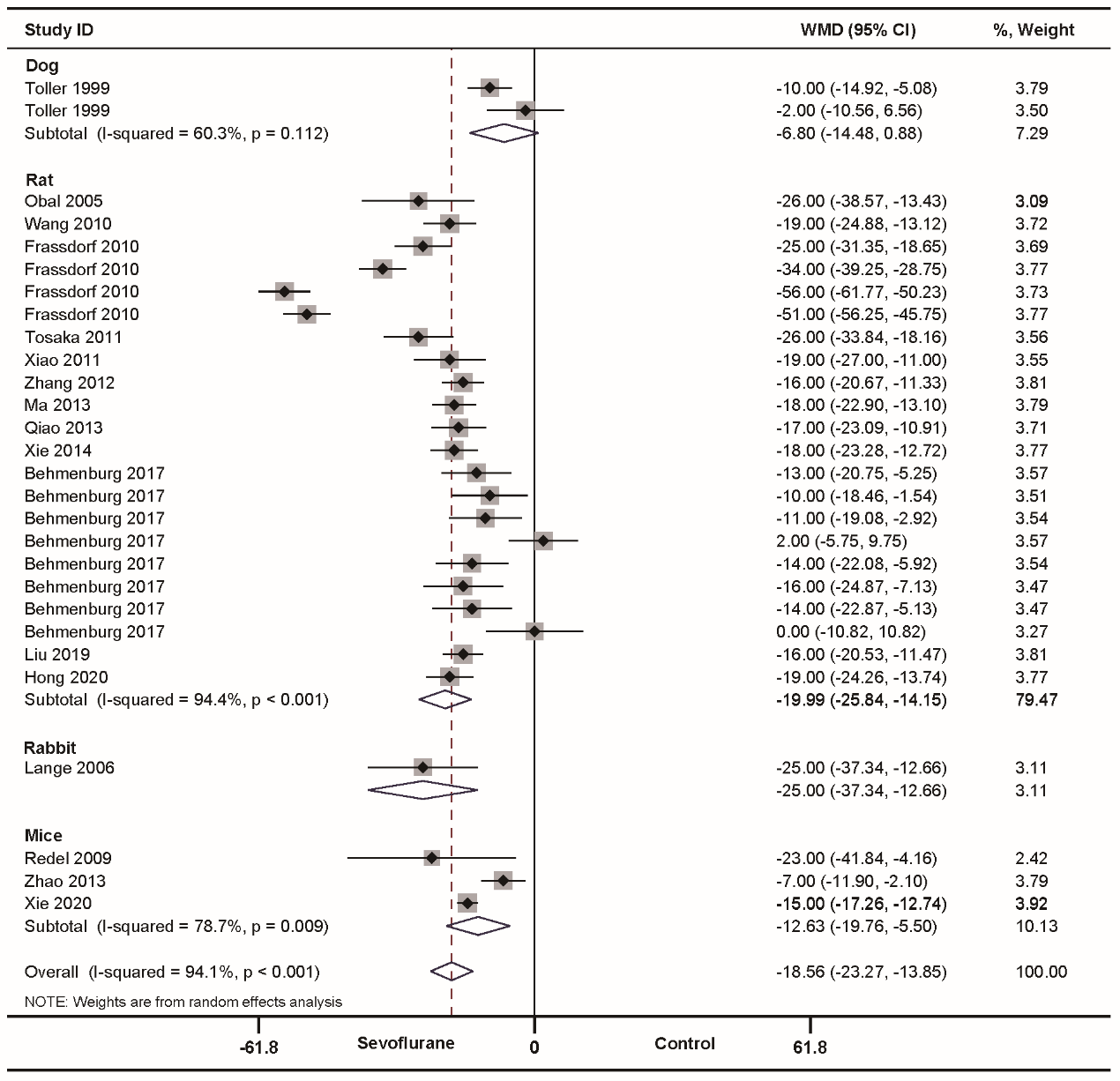


**Note：**Stratified analysis by species in SPreC group.
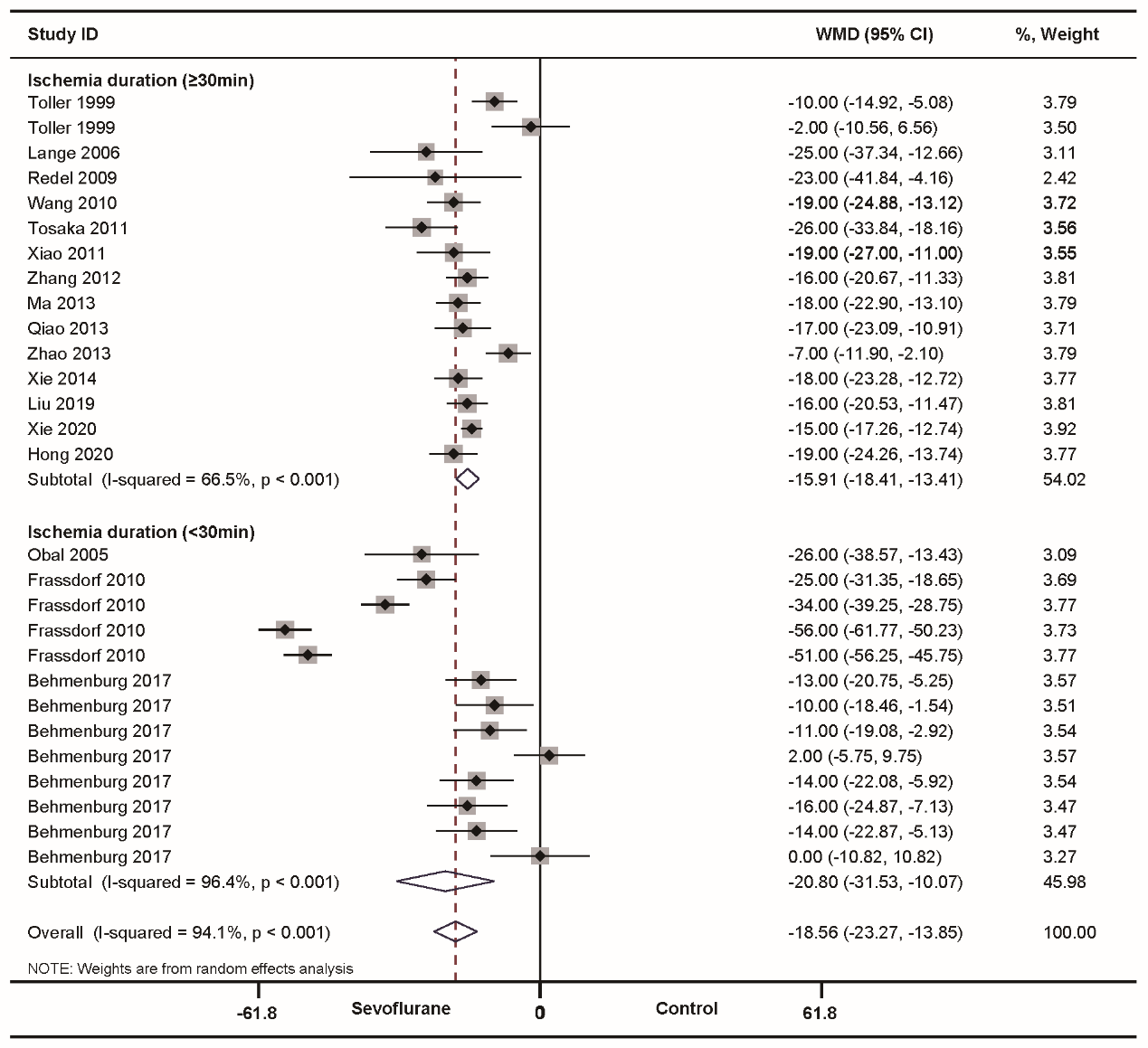


**Note：**Stratified analysis by ischemia duration in SPreC group.


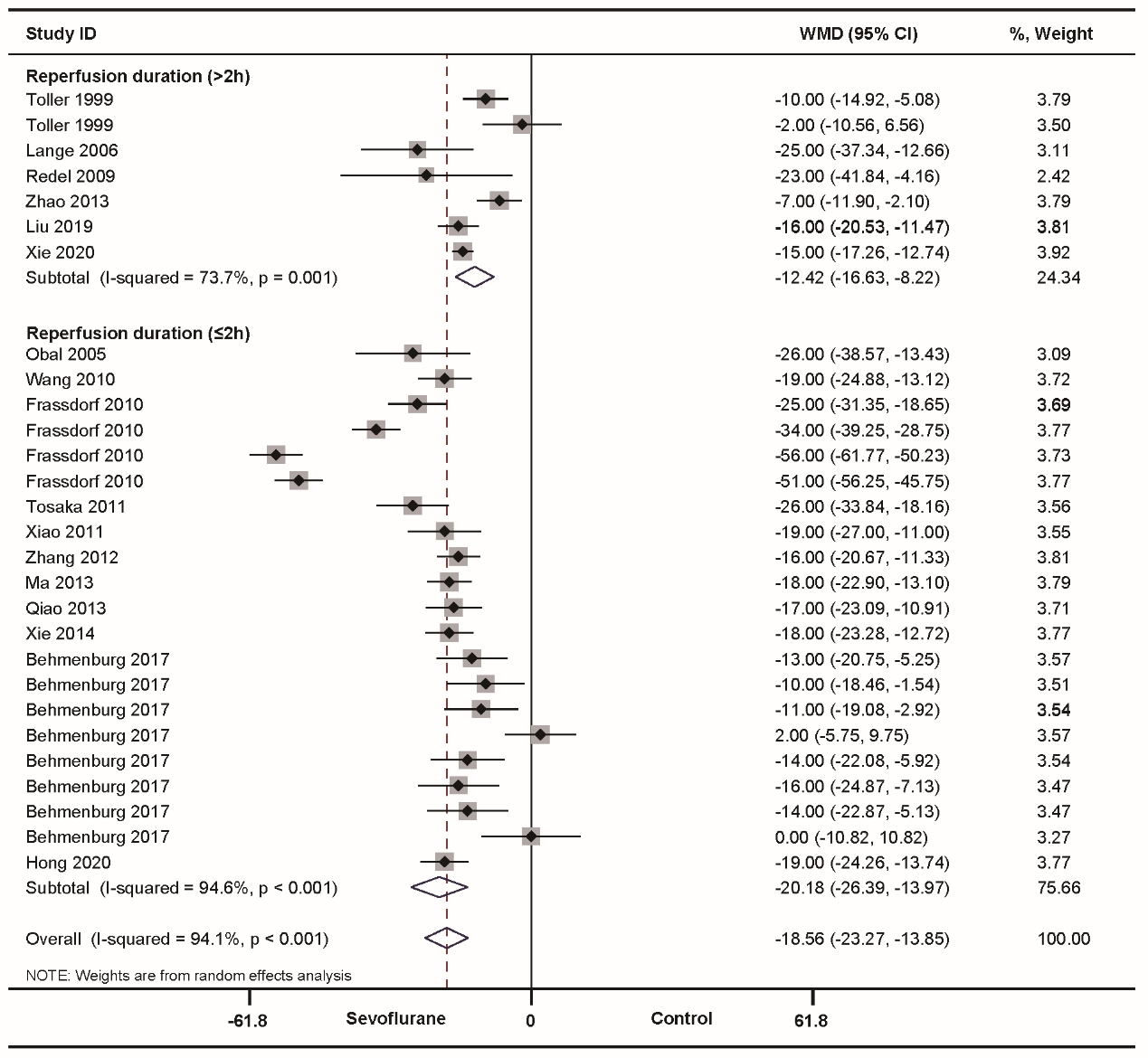


**Note：**Stratified analysis by reperfusion duration in SPreC group.


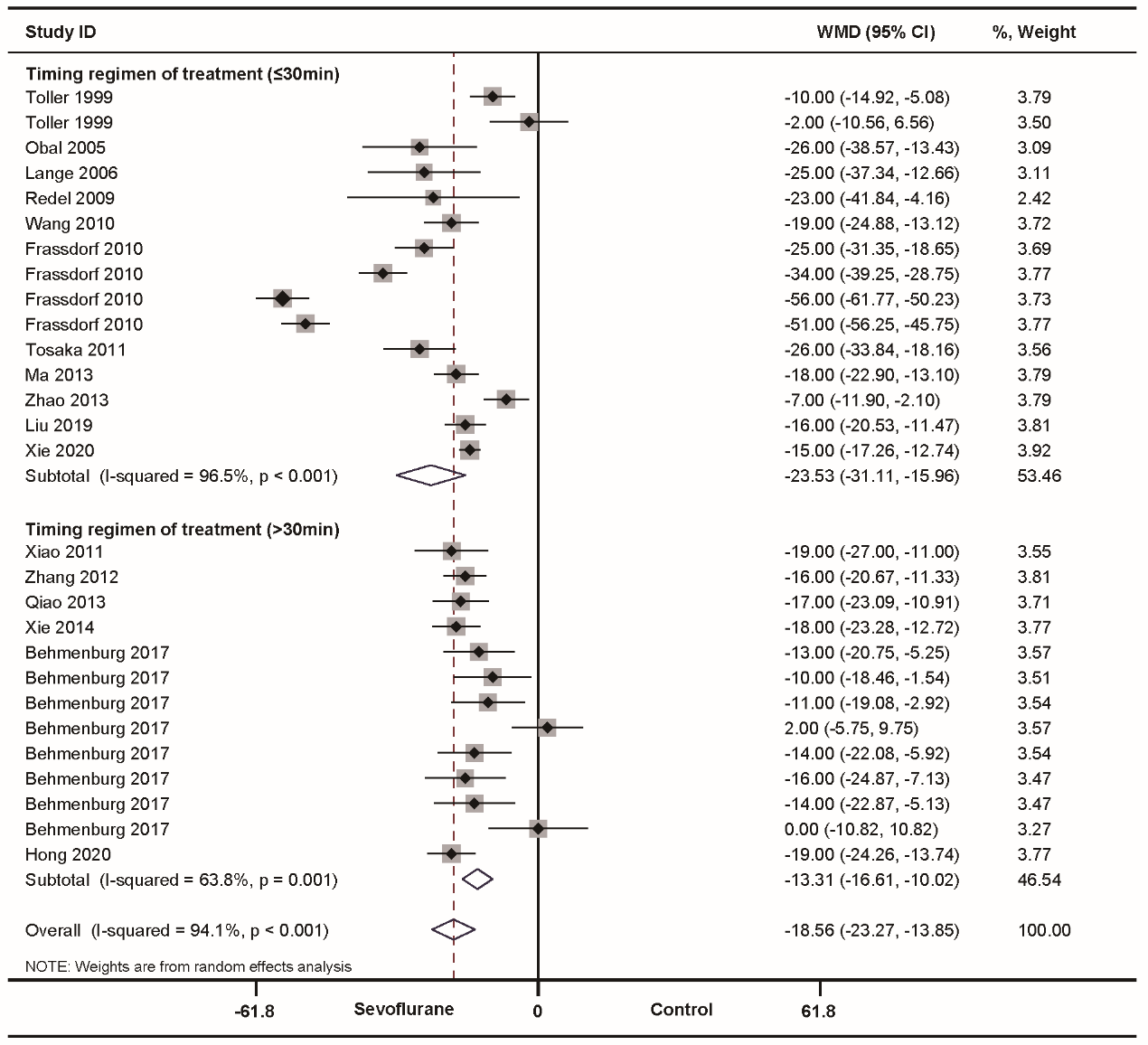


**Note：**Stratified analysis by timing regimen of treatment in SPreC group.


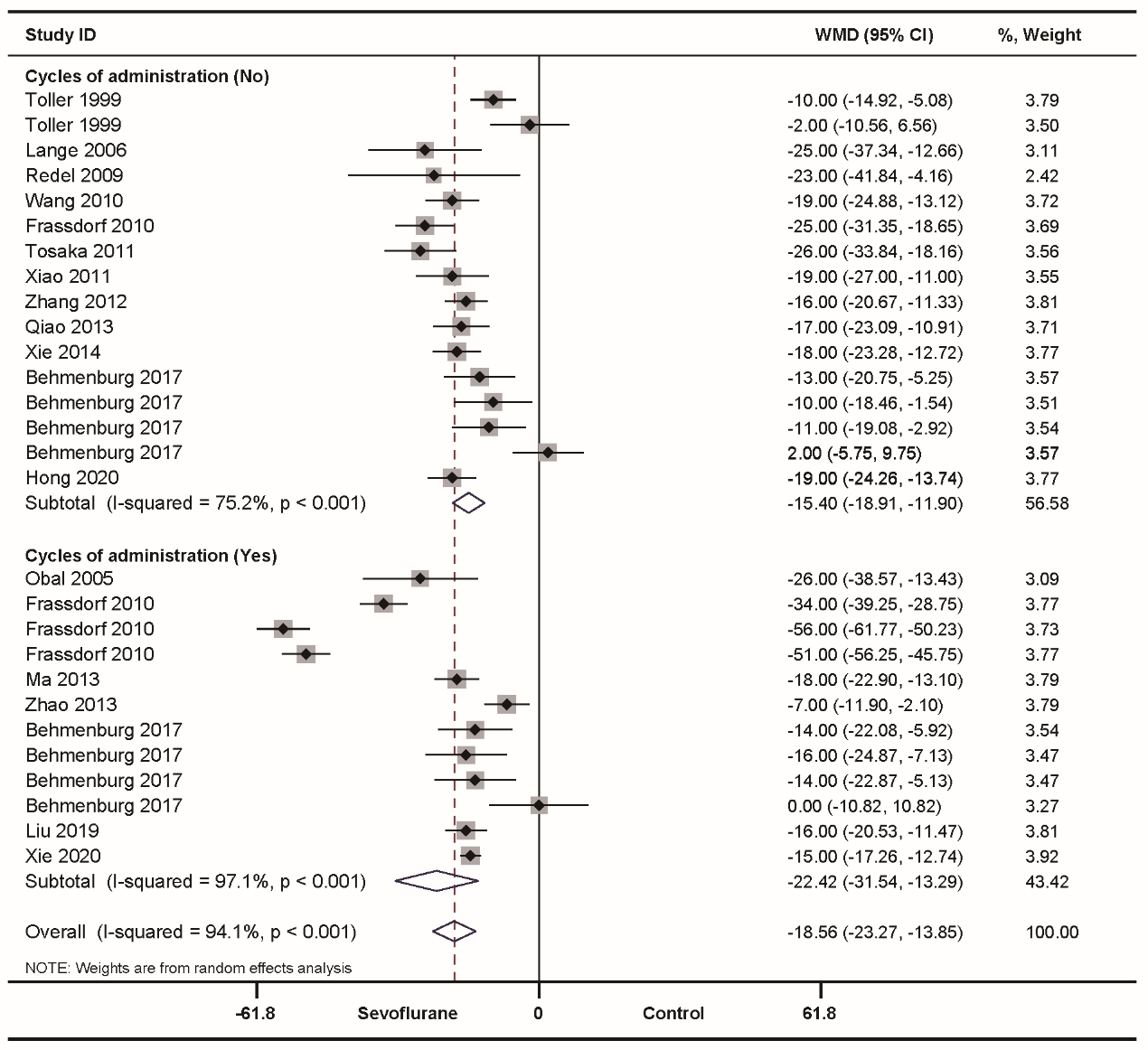


**Note：**Stratified analysis by cycles of administration (Yes/No) in SPreC group.


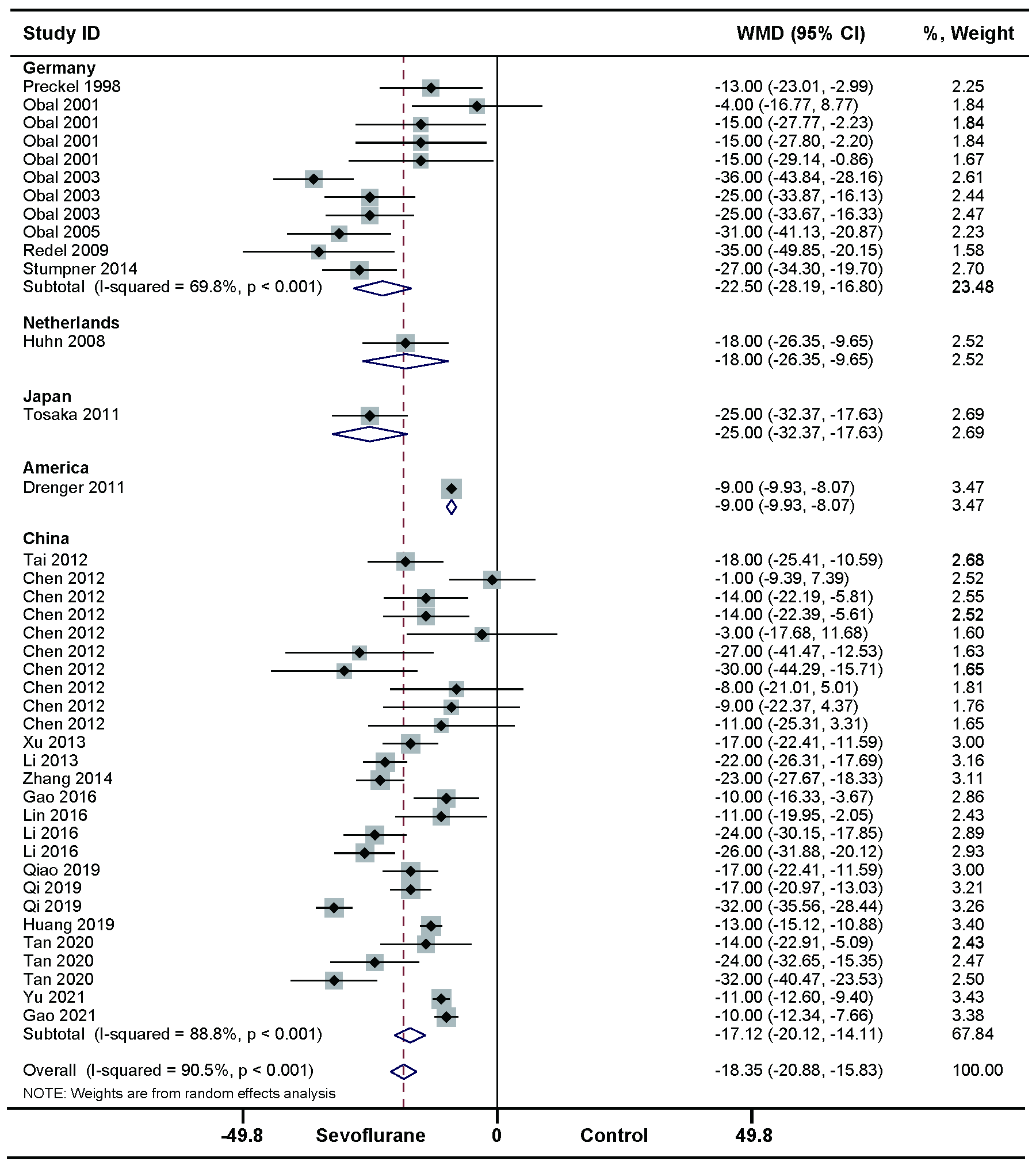


**Note：**Stratified analysis by countries in SPostC group.


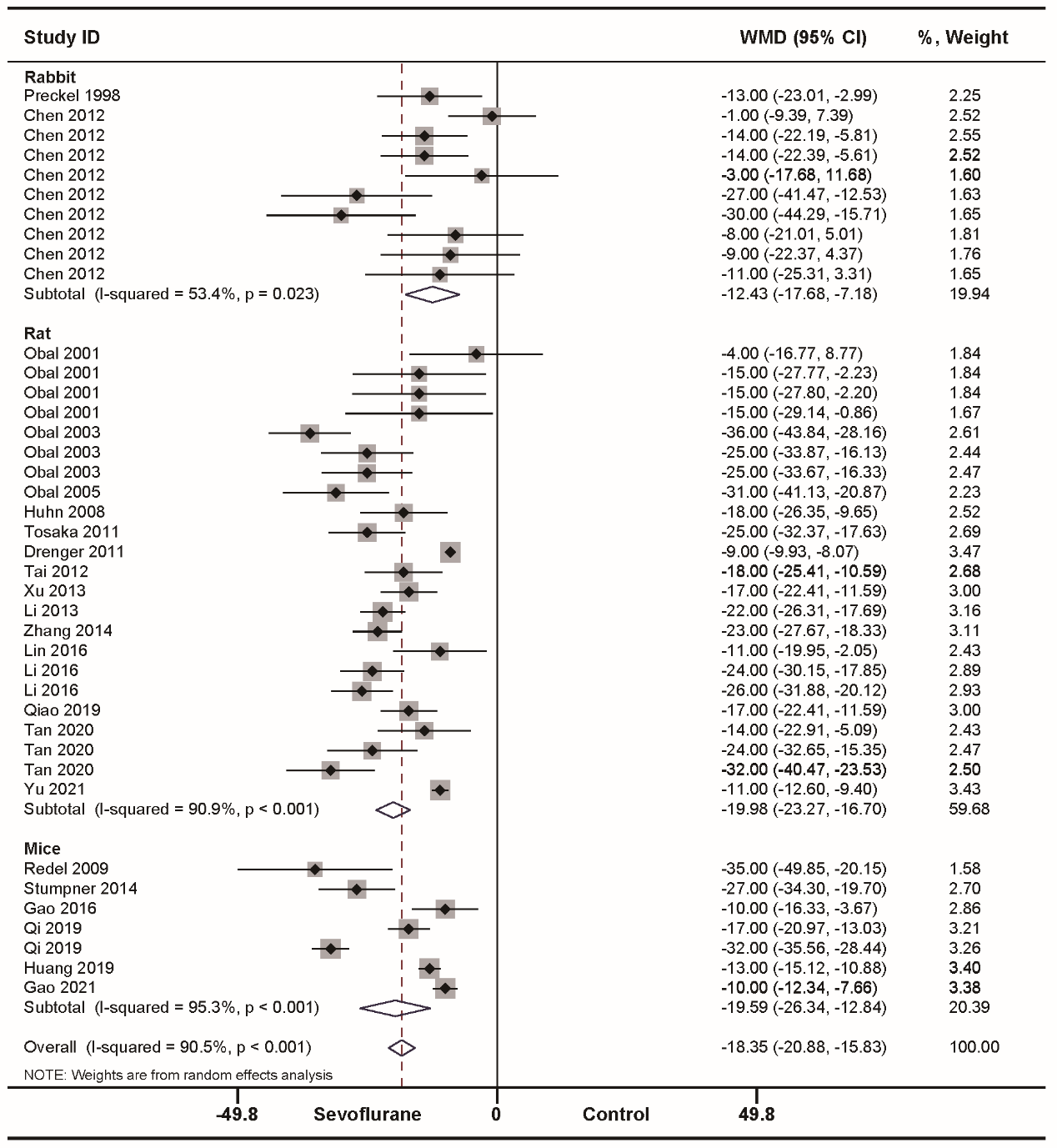


**Note：**Stratified analysis by species in SPostC group.


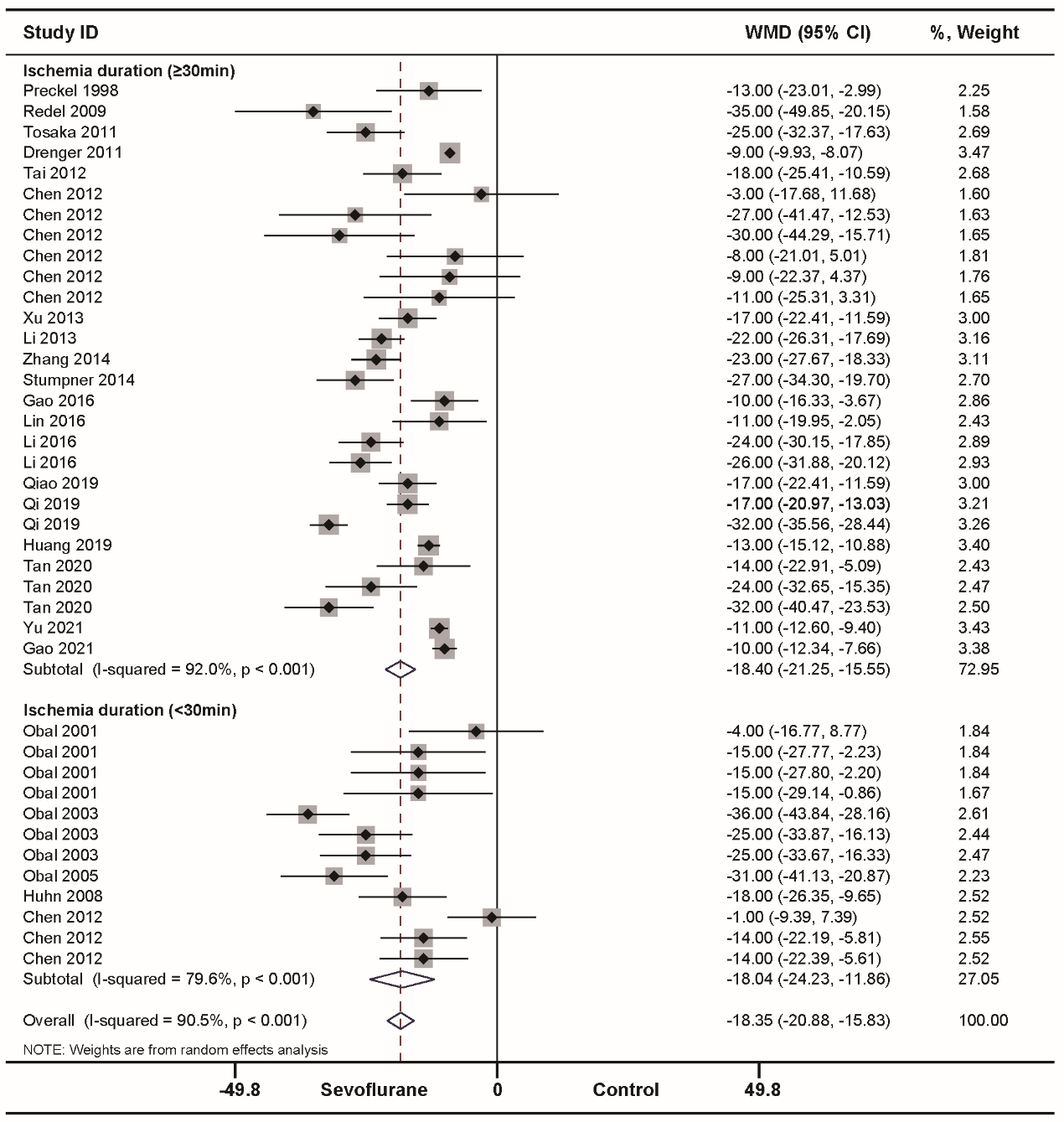


**Note：**Stratified analysis by ischemia duration in SPostC group.


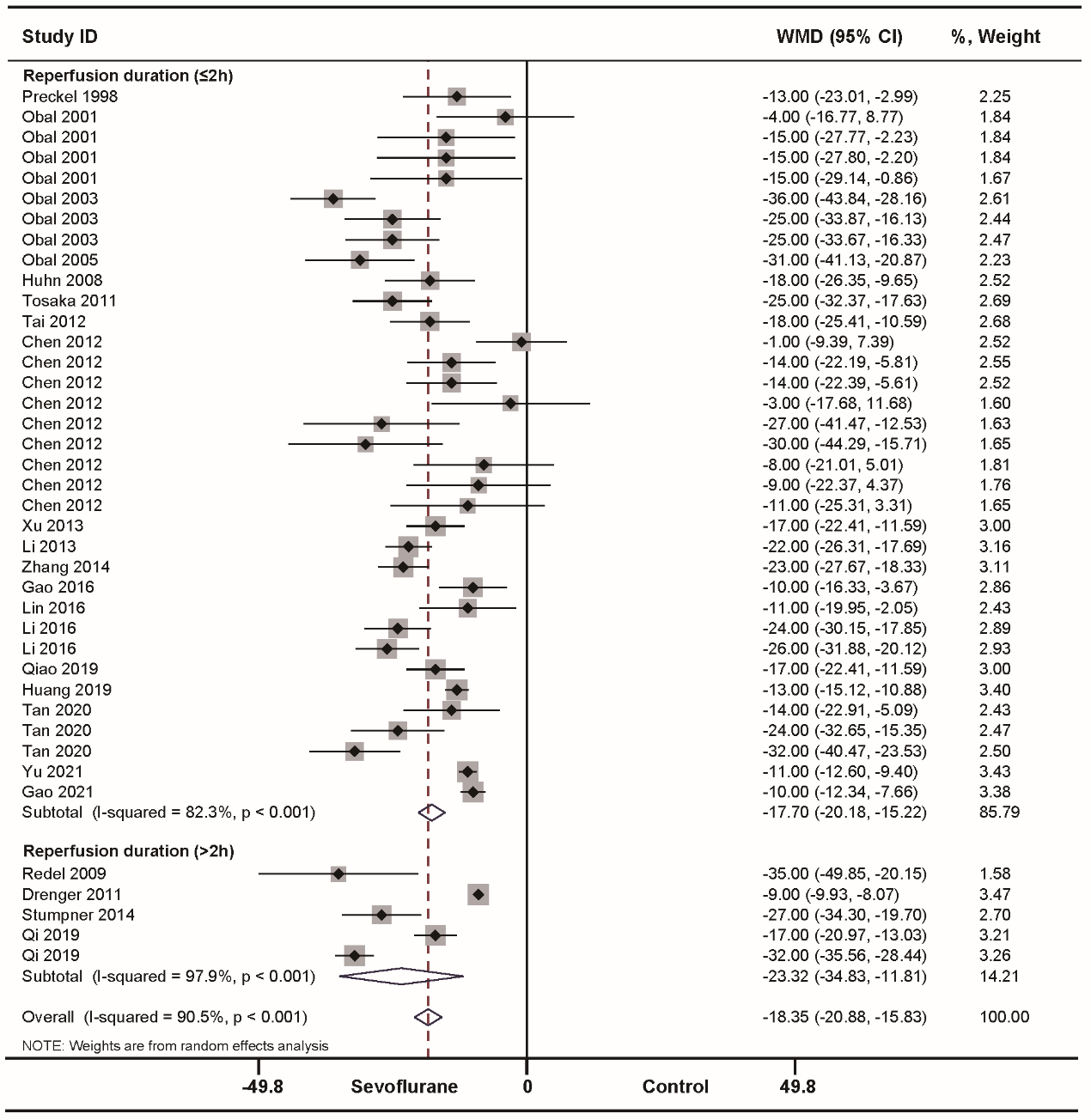


**Note：**Stratified analysis by reperfusion duration in SPostC group.


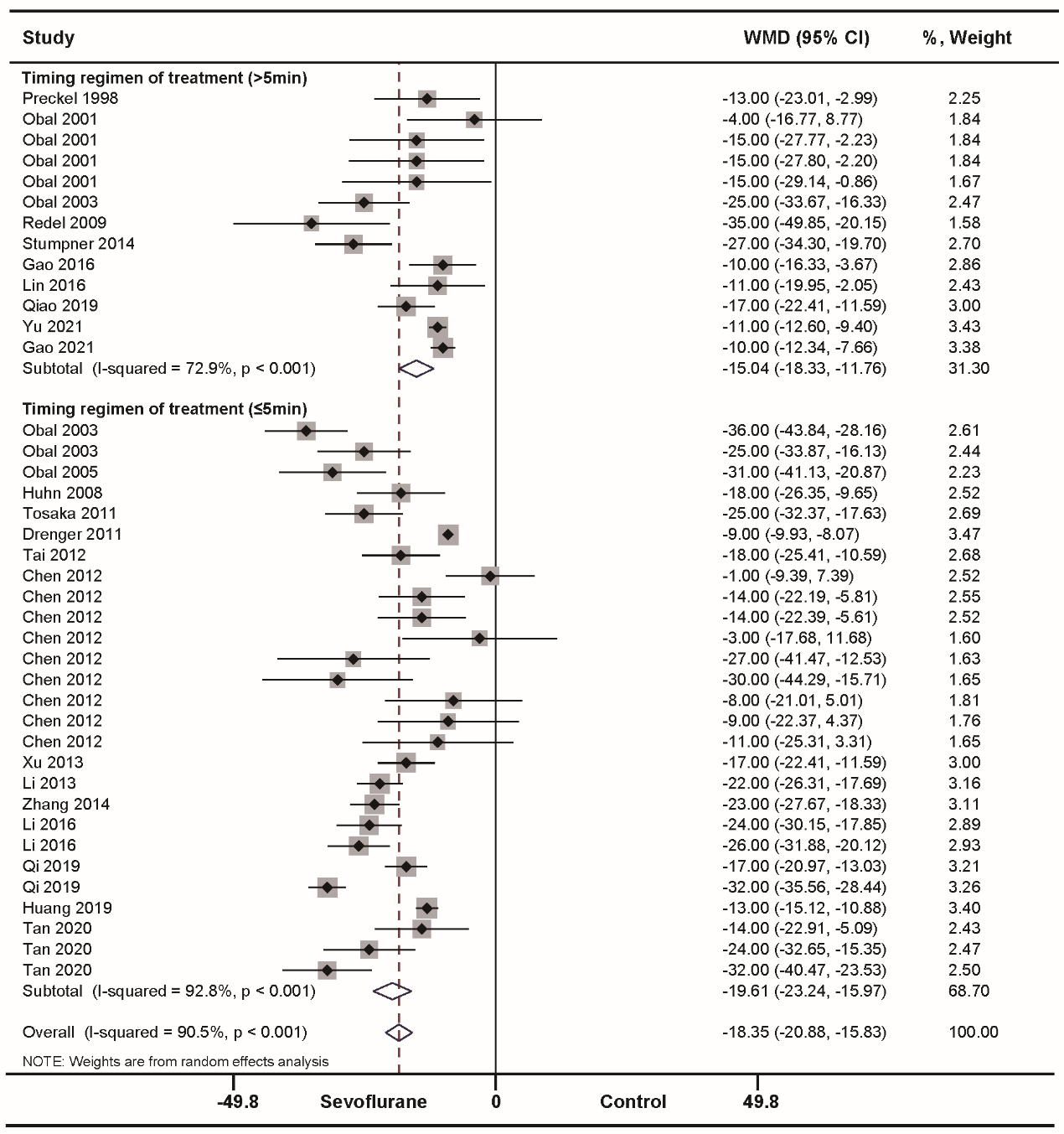


**Note：**Stratified analysis by timing regimen of treatment in SPostC group.


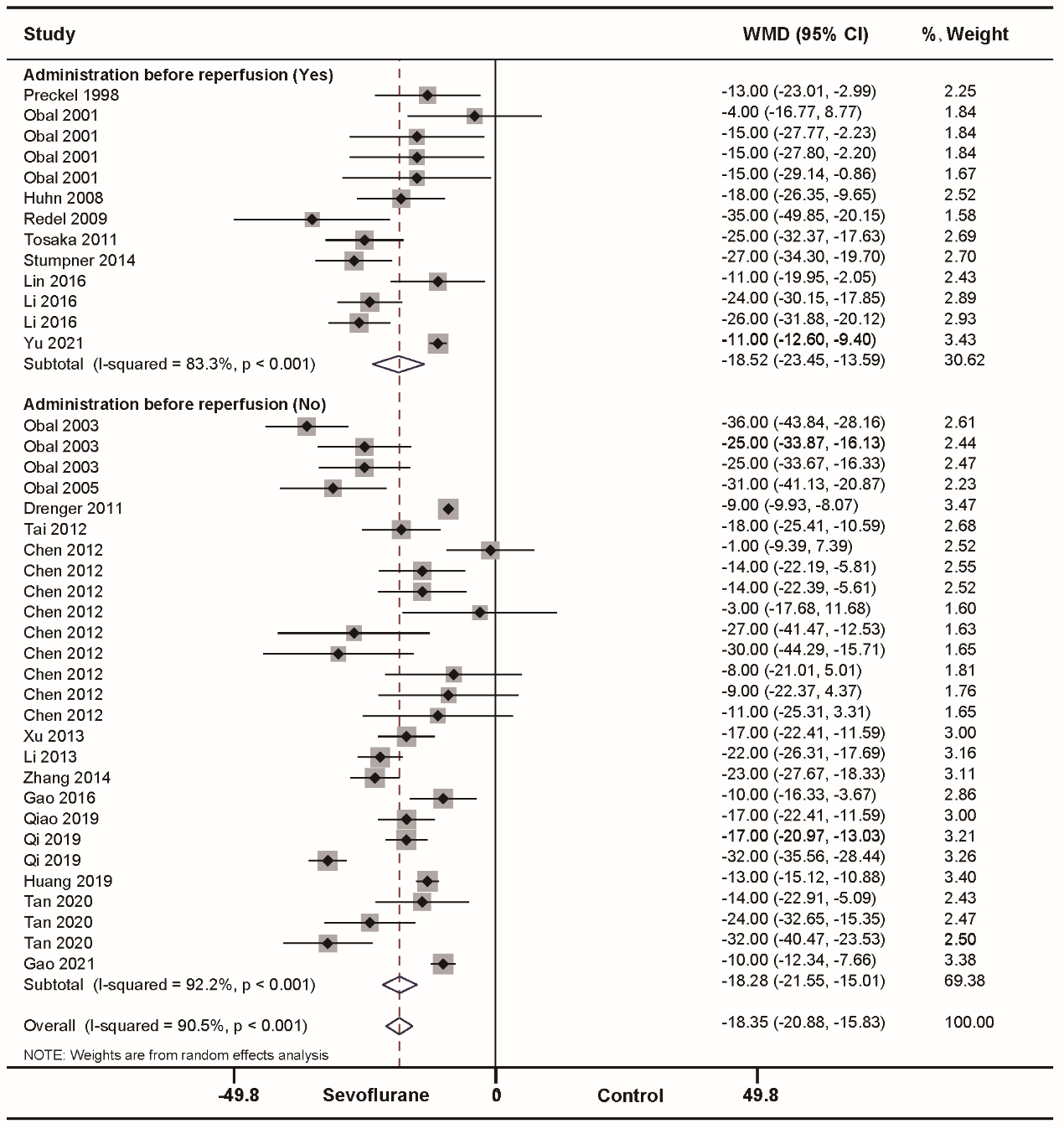


**Note：**Stratified analysis by administration before reperfusion (Yes/NO) in SPostC group.
